# Genomic Characterization and Antimicrobial Resistance Profile of *Streptococcus uberis* Strains Isolated from Cows with Mastitis from Northwestern Spain

**DOI:** 10.3390/antibiotics14111059

**Published:** 2025-10-23

**Authors:** Emiliano J. Quinto, Paz Redondo del Río, Beatriz de Mateo Silleras, Alberto Prieto, Gonzalo López-Lorenzo, Carlos M. Franco, Beatriz I. Vázquez

**Affiliations:** 1 Department of Nutrition and Food Science (Research Group on Nutrition and Microbial Dynamics), Faculty of Medicine, University of Valladolid, 47005 Valladolid, Spain; equinto@uva.es (E.J.Q.); paz.redondo@uva.es (P.R.d.R.); bmateo@uva.es (B.d.M.S.); 2 Department of Animal Pathology (INVESAGA Group), Faculty of Veterinary Medicine, Campus Terra, University of Santiago de Compostela, 27002 Lugo, Spain; alberto.prieto@usc.es (A.P.); gonzalo.lopezlorenzo@usc.es (G.L.-L.); 3 Instituto de Biodiversidade Agraria e Desenvolvemento Rural (IBADER), Campus Terra, University of Santiago de Compostela, 27002 Lugo, Spain; 4 Hygiene, Inspection and Food Control Laboratory, Analytical Chemistry, Nutrition and Bromatology Department, Faculty of Veterinary Medicine, Campus Terra, University of Santiago de Compostela, 27002 Lugo, Spain; beatriz.vazquez@usc.es

**Keywords:** antimicrobial resistance, *Streptococcus uberis*, bovine mastitis, virulence factors

## Abstract

**Background/Objectives**: *Streptococcus uberis* is a Gram-positive bacterium and a major cause of bovine mastitis. The use of antimicrobial treatments raises concerns about resistance. This study aimed to characterize *S. uberis* isolates from one of the ten largest milk-producing regions in Europe. **Methods**: Thirty-six isolates from 36 cows with mastitis were identified using MALDI-TOF and VITEK^®^MS. Susceptibility to 9 antibiotics (penicillin G, ampicillin, tetracycline, erythromycin, clindamycin, cefotaxime, ceftriaxone, levofloxacin, and moxifloxacin) was determined with VITEK^®^2. Whole-genome sequencing was performed using MinION Mk1C. **Results**: Alleles were identified for 7 loci: *arcC*, *ddl*, *gki*, *recP*, *tdk*, *tpi*, and *yqiL*. Only 10 isolates had alleles for all the loci. The loci with the highest number of alleles were *ddl* and *tdk* (33/36 strains), while *arcC* had the fewest (19/36). Four isolates were assigned to known sequence types (ST6, ST307, and ST184), and novel alleles were detected in 32 of the 36 isolates. Twelve isolates showed phenotypic resistance to one or more of the following antibiotics: tetracycline, erythromycin, clindamycin, and ceftriaxone. The *lnu* was the most frequently detected resistance gene (27 out of 102 total gene appearances). A total of 19 virulence factors were identified. All strains were predicted to be capable of infecting human hosts. **Conclusions**: *Streptococcus uberis* is a potential reservoir of antimicrobial resistance genes. The use of antimicrobials to treat bovine mastitis has reduced the susceptibility of this microorganism to several antibiotics, underscoring the importance of monitoring antimicrobial use in veterinary practice. The results also highlight the high genetic diversity of the isolates, suggesting a strong capacity to adapt to different environmental conditions.

## 1. Introduction

Owing to the H5N1 influenza outbreaks in the early 2000s, the American Veterinary Medical Association established a One Health Initiative Task Force in 2006, and the American Medical Association approved a One Health resolution in 2007 [1], establishing links between animal health, human health, and the environment. In 2008, six international organizations developed a framework titled “Contributing to One World, One Health—A Strategic Framework for Reducing Risks of Infectious Diseases at the Animal–Human-Ecosystems Interface”, aimed at achieving optimal health for humans, animals, and the environment [2].

According to the FAO, milk output in Europe reached approximately 232 million tons in 2022, and 159 million tons in the European Union [3]. The dairy sector in Galicia (northwestern Spain) is strategically important, accounting for 1.5% of the region’s GDP. In 2024, Galicia had 6170 dairy farms, producing 3.1 million tons of milk—38.6% of total Spanish milk production [4]. Galicia is among the top ten milk-producing regions in Europe. Recently, the Organization for Economic Cooperation and Development (OECD) and the Food and Agriculture Organization of the United Nations (FAO) reported several risks and uncertainties facing the milk sector, including the increased prevalence of mastitis, economic losses, and antimicrobial resistance [5].

*Streptococcus uberis* (*S. uberis*) is a Gram-positive bacterium in the *Streptococcaceae* family, which includes both commensal and pathogenic species [6,7]. Pathogenic species cause different diseases in animals, primarily bovine mastitis [8]. The zoonotic potential of some streptococci has been reported for *S. suis* and *S. agalactiae* [6,9,10]. Although *S. uberis* is rarely associated with human infections, 17 cases have been reported [11]. For example, a 9-year-old boy who sustained a frontoparietal scalp laceration after being kicked by a horse developed a low-grade fever and subcutaneous swelling 11 days into treatment; *S. uberis* was isolated from the wound fluid [12]. In another case, a 75-year-old man developed a hemorrhagic plantar bulla after frequently hiking barefoot across cow-populated pastures; the lesion progressed to a secreting ulcer, and *S. uberis* was identified [11].

*S. uberis* is associated with persistent intramammary infections in cows due to its ability to invade and persist within the mammary gland [13]. This microorganism has also developed increased resistance to antimicrobials [14,15,16,17,18], which poses health risks at the herd level [19]. In addition, various virulence factors are involved in the colonization of the mammary gland, although the complete pathogenic mechanisms of *S. uberis* remain unclear [20].

Strains isolated from bovine mastitis show high polymorphism, suggesting that the primary source of *S. uberis* is the dairy farm environment [21,22,23,24]. However, cow-to-cow transmission has also been reported [25,26,27,28,29]. In Galicia, *S. uberis* was isolated in 36.45% of clinical milk samples where the microorganism was detected, underscoring its prevalence and persistence in the dairy environment [30]. Despite its widespread occurrence in the region, little is known about the genomic diversity and resistance determinants of *S. uberis* in Galicia. Advanced molecular tools, such as whole-genome sequencing, facilitate the identification of genes related to antimicrobial resistance, virulence, and environmental adaptation [6,31,32], thus supporting the development of strategies for the prevention and control of this pathogen [32].

The present study aimed to perform a whole-genome sequence analysis of several *S. uberis* strains isolated from cases of bovine clinical mastitis in northwestern Spain. DNA sequencing was conducted using MinION technology, and bioinformatic analysis was performed using the Galaxy platform.

## 2. Results

### 2.1. Descriptive Data

**Figure 1:**
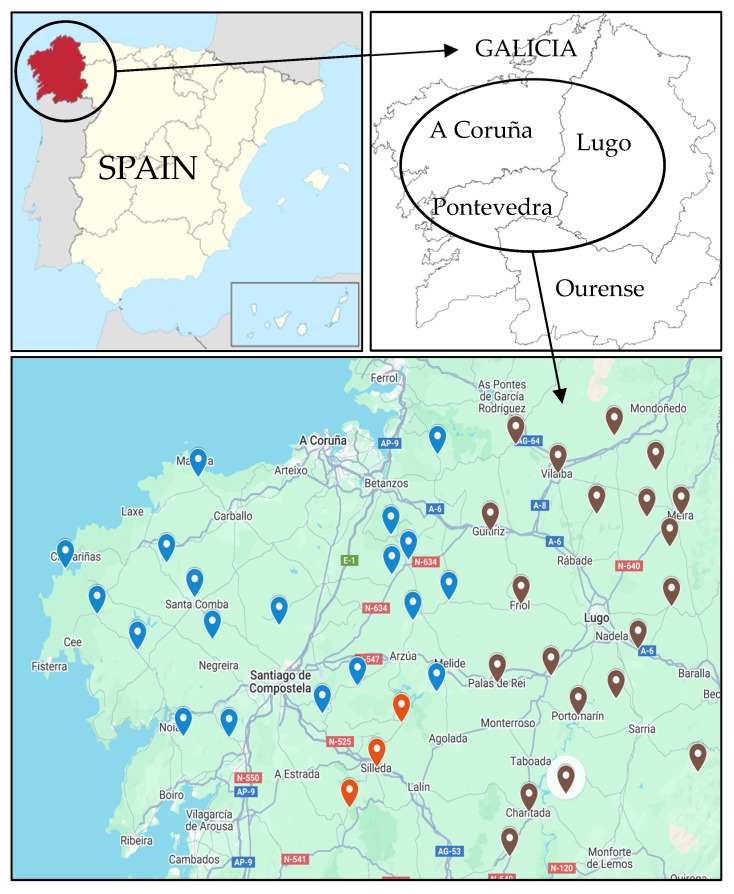
Geographical locations of the sampled dairy farms. Brown markers: Lugo province; blue markers: A Coruña province; and red markers: Pontevedra province.

A total of 36 *S. uberis* strains were selected from clinical mastitis cases on 42 dairy farms in the Galicia region (northwestern Spain; see Figure 1). Milk samples were collected from the provinces with the largest dairy cow populations in 2022—Lugo (137,662 animals), A Coruña (131,468 animals), and Pontevedra (35,258 animals) [33].

In the present study, 36 strains were successfully sequenced via the Oxford Nanopore basecalling software Guppy version 6.4.6. The sequencing statistics were generated with the Nanoplot tool from the Galaxy platform and the Geneious bioinformatics software version 2024.0 (Supplementary File S1). The following metrics were obtained from the Nanoplot tool: number of reads and bases, median, mean, and standard deviation of the read length, n50 (the length of contigs equal to or longer than half of the total assembly length), and the longest read in the first quartile. For all the strains analyzed, read counts ranged from 60,969 (strain 1121190) to 507,352 (strain 1121776), while read length ranged from 522.3 (strain 1121190) to 6212.2 (strain 1122931). The number of bases sequenced (the number of reads times the mean read length) had a maximum of 1.12 Mbp (strain 1121208) and a minimum of 31.84 Mbp (strain 1121190).

The number of sequences, percentage of bases G and C per read, and sequence length were obtained using the Geneious software version 2024.0. Genome sizes ranged from 1.88 (strain 1122603) to 2.27 Mbp (strain 1121287).

### 2.2. MLST

The results from the *S. uberis* MLST (multilocus sequence typing) database for the strains analyzed are shown in Supplementary File S2A. The table includes the loci used in the MLST scheme and the best-matching MLST alleles. Strains 1121287 and 1121774 presented perfect matches with multiple alleles in the database of the MLST tool, specifically for the alleles *arcC* and *recP*, respectively. Supplementary File S2B lists the sequence type (ST) and the closest ST for each strain as assigned by the MLST tool. Only 4 strains corresponded to a previously known STs: 1121208 (ST6), 1121227 (ST307), 1121338 (ST184), and 1122931 (ST307). Strain 1121751 was assigned to ST386, but with alleles showing less than 100% identity despite full coverage. Interestingly, strain 1121774 contained alleles with multiple perfect matches, suggesting the possibility of more than one ST, although none corresponded to the nearest STs. Alleles were identified across 7 *loci (arcC*, *ddl*, *gki*, *recP*, *tdk*, *tpi*, and *yqiL*), although only 10 out of 36 strains carried alleles for all loci. The loci with the highest allele counts were *ddl* and *tdk* (33/36 strains), while *arcC* was the least represented (19 strains). The *tdk* gene (encoding thymidine kinase) exhibited the highest variability (12 variants), followed by *ddl* (D-alanine-D-alanine ligase) and *gki* (glucokinase) with 9 variants each. *recP* (transketolase) and *yqiL* (acetyl-CoA acetyl transferase) showed the lowest variability (4 variants each). The remaining two genes, *arcC* (carbamate kinase) and *tpi* (triosephosphate isomerase), presented 6 and 5 variants, respectively.

**Figure 2:**
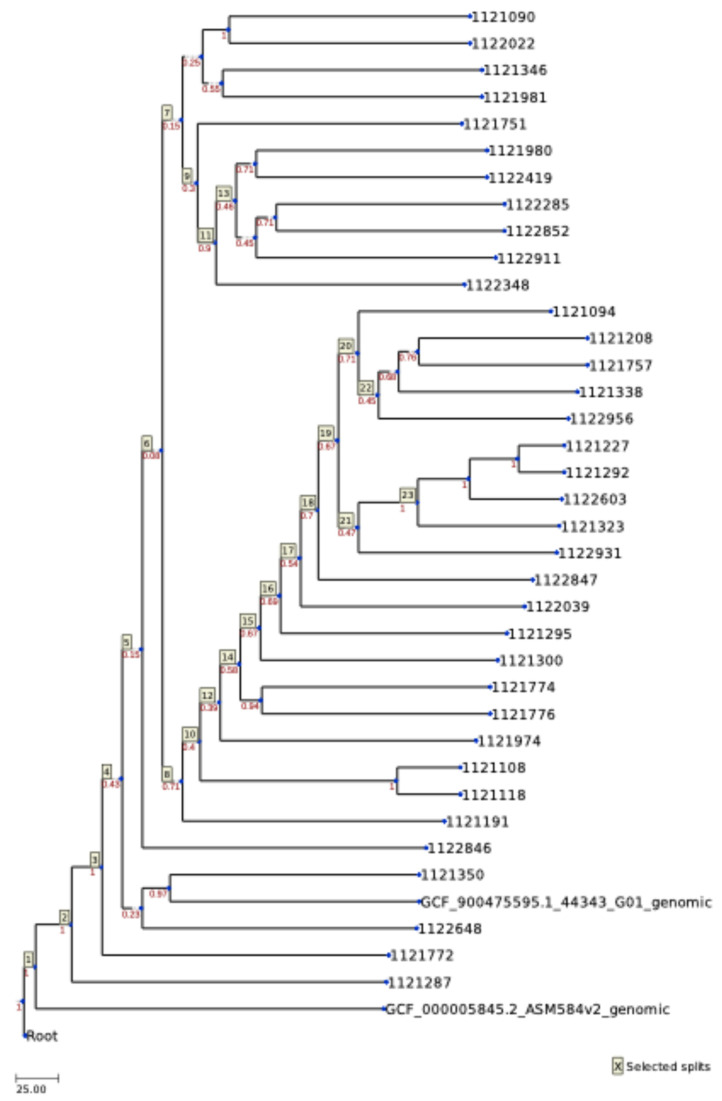
The wgMLST tree. It illustrates the genetic relatedness among the analyzed isolates based on allelic differences across the whole genome. Each node represents an individual isolate, and branch lengths correspond to the number of allele differences between isolates. Isolates closely clustered share a high proportion of identical alleles, indicating close genetic relationships and potential epidemiological linkage.

**Figure 3:**
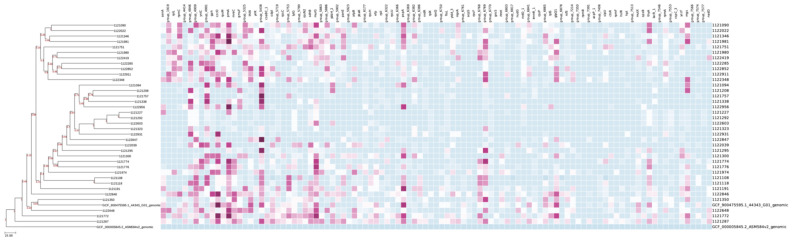
Heatmap from wgMLST (101 highly discriminatory loci; see Supplementary File S2C). Each cell represents the number of differing alleles between two genomes, with color intensity indicating the degree of genetic similarity (lighter colors represent closely related isolates, i.e., fewer allele differences; conversely, darker colors indicate greater allelic divergence).

The genomes of the 36 *S. uberis* strains, along with 2 reference strains (*S. uberis*, genome assembly 44343_G01, NCBI RefSeq GCF_900475595.1; and *Escherichia coli*, genome assembly ASM584v2, NCBI RefSeq GCF_000005845.2) were analyzed using the Cano-wgMLST_BacCompare platform. The outputs included a Venn diagram (Supplementary File S2D), a descriptive table showing the selected wgMLST (whole-genome MLST) scheme and the most discriminatory loci (occurring in >95% of isolates) (Supplementary File S2C), a genetic relatedness wgMLST tree (Figure 2), and a heatmap profile (Figure 3).

The Venn diagram illustrated the genome composition by showing loci included in 100% (Occ100), 90% (Occ90), 70% (Occ70), or 50% (Occ50) of isolates. Specifically, 0, 602, 1076, and 1483 loci were identified in 100, 90, 70, and 50% of the isolates, respectively, reflecting the expected decrease in shared loci as more strains were considered (Supplementary File S2D). Notably, the results highlight that 602 loci were present in ≥90% of isolates, indicating high genetic diversity.

The most discriminatory loci (Supplementary File S2C) indicated that 416 loci were shared by ≥95% of isolates, including 101 loci identified as highly discriminatory based on their strong ability to differentiate between isolates [34].

A wgMLST tree was also constructed (Figure 2). The topology of the tree was divided into two major groups: one containing the *E. coli* reference strain, and the other comprising all the *S. uberis* strains, including the *S. uberis* reference strain. Within the *S. uberis* branch, the reference strain clustered in the third divergence event, forming a group with strains 1121350 and 1122648, both isolated in the province of Lugo. The second branch from this divergence included all remaining isolates, except for strain 1121772 (from the province of A Coruña) and strain 111287 (from the province of Lugo), which diverged earlier. The fifth split divided the strains into two main groups, but this division did not correlate with their geographic origin. Interestingly, the two strains from the province of Pontevedra fell into separate groups: strain 1121757 clustered with one branch, while strain 1122419 clustered with the other. Figure 3 presents the heatmap of these 101 highly discriminatory loci.

To obtain the pangenome, the genomic sequences of the 36 *S. uberis* isolates were reanalyzed strains in the Cano-wgMLST_BacCompare platform, this time excluding the reference strains. The resulting pangenome comprised 10,122 genes: 521 (5%) core genes present in ≥95% of the isolates, 5691 (56%) accessory genes, and 3910 (39%) unique genes specific to individual strains. The corresponding Venn diagram, wgMLST tree, most discriminatory loci, and heatmap profile are provided in Supplementary File S3A–D.

### 2.3. Antimicrobial Profile

The phenotypic antimicrobial profiles and resistance genes of the *S. uberis* strains are summarized in Table 1. Twelve of the 36 strains (33.3%) showed phenotypic resistance to one or more of the following antibiotics: tetracycline (TET), erythromycin (ERY), clindamycin (CLI), and ceftriaxone (CRO). Although no strain was resistant to all four antibiotics simultaneously, all resistant strains (12/36) exhibited resistance to CLI. Three strains showed phenotypic resistance to TET, ERY, and CLI, while only one strain showed resistance to CRO. Four strains did not display any phenotypic antimicrobial resistance.

The resistance genes are also listed in Table 1 and classified by frequency of occurrence in Table 2. The *lnu* gene was the most common, with 27 occurrences out of 102 total hits, demonstrating concordance between phenotypic and genotypic resistance. Only three strains carried two *lnu* genes simultaneously: 1121772, 1122852, and 1122911. The *mph14* gene was the least frequent (3/102). The predicted antimicrobial resistance phenotypes for all *S. uberis* strains are provided in Supplementary File S4.

### 2.4. Plasmids and Virulence

Plasmid content was assessed using the PlasmidFinder tool with default thresholds (95% identity and 60% coverage). Only 10 strains showed alignment to a plasmid sequence, and all matched a single plasmid replicon: *repUS43* (Supplementary File S5A). This replicon initiates plasmid DNA replication and has been associated with resistance to beta-lactamases, aminoglycosides, and tetracycline. The *repUS43* plasmid is also found in mobilizable plasmids, which are capable of being transferred to other bacteria via conjugation [35]. The query/HSP length was identical: 1206/1206, with *Rep_trans* identified as the targeted locus.

Virulence factors (VF), defined as gene products (e.g., toxins, cell surface attachment proteins, enzymes) that contribute to the pathogenicity, were identified using the VFDB. Table 3 lists the VF detected in the studied strains, grouped by major functional classes. Among the categories identified were adherence, enzymes, immune evasion, manganese uptake, proteases, cell surface components, and serum resistance. Additional details, including VF coverage, functional roles, and mechanisms in each *S. uberis* strain, are provided in Supplementary File S5B.

Finally, Supplementary File S5C summarizes the predicted pathogenic potential of the isolates toward human hosts, using the PathogenFinder tool. In silico analysis predicted all *S. uberis* isolates to have human pathogenic potential.

## 3. Discussion

This study aimed to identify the genetic diversity among 36 strains of *S. uberis* previously isolated from bovine mastitis in northwestern Spain. *S. uberis* is one of the main pathogens responsible for bovine mastitis [36], causing estimated annual global losses of up to 125 billion Euros [8,36]. Although several strains have been typed by MLST, only 187 *S. uberis* genomes are currently available in the NCBI database. Consequently, prophylactic vaccine development remains under investigation, as knowledge about the bacterium is still limited [36,37].

The genome sizes of the isolates ranged from 1.9 to 2.3 Mbp, and GC content varied between 36.3 and 36.8%, consistent with previously reported values [36,38]. The NCBI database also shows comparable ranges (1.6–2.5 Mbp, GC content 36.0–37.0%).

Different gene expression patterns enable *S. uberis* to adapt to changing environmental and stress conditions [6]. Accurate strain identification is therefore essential, not only for understanding these adaptations but also for guiding epidemiological surveillance and public health interventions [39]. MLST, based on the sequencing of internal fragments of 6–7 housekeeping genes (approximately 450–500 bp each), provides a robust method for characterizing bacterial isolates [40]. Although this represents only a fraction of the genome, it is considered representative of overall genomic diversity [39,41,42].

In our study, a high proportion of novel alleles (32/36 strains) were detected, indicating considerable genetic diversity that likely reflects environmental conditions rather than clonal spread. Alleles were identified across 7 *loci (arcC*, *ddl*, *gki*, *recP*, *tdk*, *tpi*, and *yqiL*). The loci with the highest allele counts were *ddl* and *tdk* (33/36 strains), while *arcC* was the least represented (19 strains). The *tdk* gene exhibited the highest variability (12 variants), followed by *ddl* and *gki* with 9 variants each. These findings align with Fenske et al. [36], who also identified *tdk* as highly variable; the pubMLST database listed a high number of records for this gene (175). The *ddl* and *gki* genes also have a high number of records or variants in the database: 96 and 110, respectively. Interestingly, the pubMLST database shows a high number of records for the gene *yqiL* (111), in contrast with its low variability in the present work. These findings suggest that these housekeeping genes are not as stable as initially thought. Fenske et al. [36] reported similar conclusions.

Despite high allelic diversity, only 4 strains matched 3 known sequence types (STs): ST6, ST184, and ST307. Strain 1121751 was related to ST386 but showed alleles with less than 100% identity despite full coverage, suggesting polymorphic variants. Strain 1121774 was classified as ST815, with multiple perfect allele matches. Overall, 32/36 strains carried novel alleles, reflecting a high level of diversity similar to findings from Germany (17/24 novel) and Australia (27/27 novel) [36,38]. Tomita et al. [43] also observed wide diversity, identifying 33 STs among 46 isolates, with ST60 and ST155 being the most common. Despite the small size of the sample, our study revealed a great diversity of allelic profiles or STs, indicating that the spread of *S. uberis* in northwestern Spain is more heterogeneous than clonal [15,36], with no clear correlation between STs and geographic location (i.e., farms in the interior vs. farms closer to the seacoast).

Single-genome sequencing provides limited insight into the genetic variability that drives pathogenicity and vaccine target identification [44,45,46]. Like *Streptococcus agalactiae*, *S. uberis* exhibits an open pangenome, consisting of a core genome (genes present in all strains) and a dispensable genome (genes absent from one or more strains—or accessory—, and genes specific to each strain). Our dataset contained 10,122 genes: 5.1% core, 56.2% accessory, and 38.6% unique. This indicates high intraspecies variability and global diversity, consistent with other studies [36,47]. Fenske et al. [36] reported smaller pangenomes (2508 genes) but larger core genomes (1611 genes). These values differ from those reported by other authors [37,38,48]. Some authors have noted that new unique genes are detected as more strains are sequenced [44].

The observed diversity may also be influenced by sequencing technology. Although High Accuracy Calling (HAC) has been used for basecalling, and assembly correction has been performed with the Medaka tool, sequencing errors inherent to Nanopore sequencing technology could affect allele identification. New flow cells, advanced pore design, and AI-driven neural networks, such as Guppy, dramatically improve the consensus accuracy of ONT up to ≥99.9% after polishing [49]. The detection of novel alleles in 32 isolates strongly suggests substantial natural diversity. Environmental factors may also play a role, given the large geographic area covered by the sampling sites across Lugo, A Coruña, and Pontevedra provinces, which encompass distinct ecological conditions.

The most common method for pangenome estimation is to include only genes present in 100% of isolates, but this approach has several limitations [50]. Any set of isolates represents only a subset of the population; if this subset lacks genetic diversity, the estimated number of core genes will be higher than in a more diverse dataset. In the present study, we defined core genes as those present in ≥95% of isolates. Using this threshold, only 5% of the pangenome was classified as core, while a larger proportion (56%) consisted of accessory genes, suggesting strong adaptive potential. Recent studies in *Streptococcus pneumoniae* have shown that core genes can exhibit higher recombination rates than accessory genes at the population level [51]. While core genes encode essential functions shared across strains, accessory genes often provide niche-specific adaptations. In our dataset, the abundance of accessory genes points to enhanced adaptability to the udder environment, where microbial communities interact and compete during mastitis [52].

Notably, novel alleles were identified in 32 of the 36 isolates, underscoring the high genetic diversity of these strains. Sequencing methodology is also a source of variability among studies. The workflow with R9.4.1 flow cells and the Rapid Barcoding Sequencing Kit enabled rapid library preparation and multiplexing of isolates, producing long-read data. In addition, the wgMLST is a whole-genome multilocus sequence typing that extends MLST to the genome level, and it has been successfully applied to several species of bacteria [53]. Homologous recombination analyses have shown that *S. pneumoniae* and other species (*Escherichia coli*, *Shigella flexneri*, *Neisseria meningitidis*, and *C. jejuni*) display relatively high recombination rates in core genes but low mutational divergence, with mutations driving the genetic differentiation underlying speciation [51,54,55]. Similarly, high mutation rates have been reported in other complex environments, such as mouse gut commensals [56]. Microbial evolution of *Escherichia coli* demonstrates that beneficial mutations can coexist with slightly deleterious ones, maintaining long-term intraspecies diversity.

Bacteria live in rich and dynamic ecosystems—such as the udder or gut—where multiple interspecies interactions shape evolution. However, little is known about how bacterial evolution unfolds in natural environments [56,57]. In this context, it is important to note the wide geographical range of the sampled provinces—Lugo (9856 km^2^), A Coruña (7950 km^2^), and Pontevedra (4495 km^2^)—which may significantly contribute to the observed variability among isolates.

*S. uberis* is a significant public health concern, as it may serve as a reservoir of antimicrobial resistance genes that can be transferred to other bacteria, including pathogens and commensals [58,59]. Antimicrobial treatment of bovine mastitis has reduced the susceptibility of this microorganism to penicillin, macrolides, aminoglycosides, and clindamycin [60]. Although antibiotics remain the most effective therapy for mastitis, monitoring the antimicrobial susceptibility of *S. uberis* is essential for effective veterinary treatment. Moreover, the antimicrobial resistance phenomenon in dairy cattle poses a potential risk to human health [59,61]. Importantly, *S. uberis* is also the most frequently isolated pathogen in sheep and goat milk [62], highlighting the importance of its surveillance in antimicrobial stewardship programs within veterinary practice.

Over the last decade, Category B antibiotics (fluoroquinolones and third- and fourth-generation cephalosporins) were among the most commonly used treatments in Spain. However, the implementation of the National Plan Against Resistance to Antibiotics (PRAN) and the adoption of electronic prescriptions have led to a marked decline in their use. At present, Category B antibiotics are reserved exclusively for cases of hyperacute mastitis caused by Gram-negative bacteria, while penicillins and sulfonamides are now the most frequently administered antimicrobials [63]. Our results show only one out of 36 strains with a phenotypic antimicrobial profile for ceftriaxone (beta-lactams and cephalosporines of third generation); these results could indicate that the PRAN has been applied in the veterinary clinical practice, reducing the number of resistant strains to this group of antibiotics.

Boireau et al. [64] conducted a ten-year study in France and reported that antibiotic resistance levels of *S. uberis* were low for gentamycin (approximately 2%), but higher for tetracycline, lincomycin, and erythromycin (20%). They also observed no seasonal variation in antimicrobial resistance across the study period. Regarding multidrug resistance, 14.5% of the *S. uberis* isolates met this criterion, while 33% were resistant to one or two antibiotic classes. In our study, 9 strains exhibited a phenotypic antimicrobial profile resistant to 2 or 3 antibiotics, specifically tetracycline, erythromycin, and clindamycin, while one strain showed resistance to tetracycline, erythromycin, and ceftriaxone. Two additional strains displayed resistance to only one antibiotic, clindamycin. In relation to other animals, Rosa et al. [65] investigated the antimicrobial susceptibility profiles of *S. uberis* isolated from sheep milk and reported that 97% of the isolates (120/124) were resistant to at least one of the 14 antibiotics tested, including erythromycin (genes ermB and ermC) and tetracycline (genes tetM, *tet*O, *tet*K, and *tet*S). Interestingly, genes such as *lnu*, *ant*, *lsa*, *aph*, and *mph* were not detected in their study, which contrasts with our findings.

The *lnu* gene was the most frequently detected, accounting for 27 out of 112 resistance genes identified. Resistance to lincosamides arises from inactivation by lincosamide nucleotidyl-transferase enzymes encoded by *lnu* genes. Six variants of the *lnu* gene have been described in the literature*—lnu*(A), *lnu*(B), *lnu*(C), *lnu*(D), *lnu*(E), and *lnu*(F). Among the strains analyzed in this study, three variants were detected: *lnu*(B), *lnu*(C), and *lnu*(D).

A total of 19 VFs and their associated genes were identified (Table 3). Five genes—*fbp54*, “undetermined”, *psaA*, *cppA*, and *htrA/degP*—were present in all the strains. The “undetermined” gene was linked to the capsule protein within the immune evasion VF class. An additional undetermined gene, associated with the agglutinin receptor protein and classified under the adherence VF class, was detected in 5 strains (15.15%). The least frequently detected genes were *srtC* (strain 1121300; related to the rlrA islet protein, adherence VF class) and *galE* and *wbtP* (strains 1121757 and 1122603; associated with the exopolysaccharide *Hemophylus* protein and the LPS *Francisella* protein, respectively).

Although *S. uberis* is rarely associated with human infections, several cases have been reported. Eight cases involved direct contact with cows, milk, and/or dairy products [66,67]. In contrast, 9 cases were not linked to cattle or milk [11,12,68,69,70,71,72,73], although 2 of them may have been indirectly related: a 9-year-old boy kicked by a horse in a field recently grazed by cattle and sheep [12], and a 75-year-old man who frequently hiked barefoot across pastures densely populated with cows [11]. Some authors have suggested that environmental factors may enhance the pathogenic potential of *S. uberis* in humans, as has been observed for *Streptococcus agalactiae* [74]. Overall, the reported cases suggest that infection with *S. uberis* may result not only from direct contact with the microorganism but also from exposure to cattle feces and/or indirect contact with contaminated pastures or farm soil.

## 4. Materials and Methods

### 4.1. Isolates and Strain Identification

Thirty-six *S. uberis* isolates were included in the study. All the strains were isolated from 36 different clinical-bovine mastitis cases detected in 42 different dairy farms in Galicia (NW Spain; ETRS89/UTM Zone 29) from the 9th to the 18th of July of 2022. Milk samples were transported under refrigeration conditions to the Galician Professional Laboratory for Milk Analyses (Laboratorio Interprofesional Gallego de Análisis de Leche—LIGAL, 15318 Abegondo, Coruña, Spain) for analysis. The MALDI-TOF (matrix-assisted laser desorption/ionization time-of-flight) mass spectrometry methodology with VITEK^®^MS (Biomérieux, Madrid, Spain) was used for strain identification.

### 4.2. Susceptibility to Antibiotics

The isolates were studied for their susceptibility to 9 common antibiotics (penicillin G, ampicillin, tetracycline, erythromycin, clindamycin, cefotaxime, ceftriaxone, levofloxacin, and moxifloxacin; Merck-Millipore, Darmstadt, Germany) via the VITEK^®^2 (Biomérieux, Madrid, Spain). The susceptibility testing (AST) cards used for the analyses are compliant with EUCAST/CLSI guidelines.

### 4.3. DNA Isolation

The isolates were transferred to our laboratory in cryovials and stored at −20 °C. For DNA isolation, a cryoball from frozen stocks was transferred to a flask containing 10 mL of BHI (Merck-Millipore, Darmstadt, Germany) and incubated at 37 °C for 18 h at 150 rpm. Then, 100 μL of the growth media was transferred to a new flask containing 10 mL of BHI and incubated under the same conditions described previously for 12 h. After that, 1 mL was transferred to a 1.5 mL microtube and centrifuged at 16,000× *g* for 2 min. The supernatant was discarded, and the DNA was isolated from the pellet following the manufacturer’s Gram-Positive Cell Lysate protocol for the Purelink Genomic DNA Mini Kit (Invitrogen^TM^, Thermo Fisher Scientific, Waltham, MA, USA). In the final step, the DNA was eluted in 50 μL of elution buffer. The DNA concentration was determined with a Qubit^TM^ fluorometer (Invitrogen, Thermo Fisher, Waltham, MA, USA) via the Invitrogen™ Qubit™ dsDNA BR Assay Kit (Invitrogen, Thermo Fisher). The DNA was stored at −20 °C until use.

### 4.4. Whole-Genome Sequencing

A concentration of 200 ng of DNA was used for sequencing with the Rapid Barcoding Sequencing Kit (SQK-RBK004, Oxford Nanopore Technologies Ltd., ONT, Oxford, UK) in combination with Flow cells R9.4.1 (ONT). MinION Mk1C software 2024.0 with integrated and preinstalled base calling and analysis software was used for sequencing. The time of sequencing was set at 24 h. Oxford Nanopore preloaded basecalling software Guppy in high accuracy (HAC) mode was used with default parameters for basecalling. Data quality control was performed as follows (see below for details): (i) a recommended coverage of 30× for bacterial genomes using NanoPlot tool; (ii) assembly and polishing with Medaka tool; and (iii) assembly with Raven tool, which performs two-step polishing with Racon tool.

### 4.5. Bioinformatic Analysis

#### 4.5.1. Assembly

De novo assembly was performed on the Galaxy platform through its European server (https://usegalaxy.eu; accessed on 1 February 2024) [75]. FASTQ files from every strain were uploaded to the Galaxy server and transformed into FASTA files via the “FASTQ to FASTA converter” tool (Galaxy Version 1.1.5) [76]. To merge all the obtained FASTA files from every strain into a unique file, the “Merge.files Merge data” tool (Galaxy Version 1.39.5.0) [77] was used. The “NanoPlot—plotting suite for Oxford Nanopore sequencing data and alignments” tool (Galaxy Version 1.41.0+galaxy0) [78] was used with the FASTQ files to create a statistical summary of the sequencing reads and to calculate the next-generation sequencing (NGS) coverage. FASTA files were used for the assembly via the “Raven—de novo assembly of Oxford Nanopore Technologies data” tool (Galaxy Version 1.8.0+galaxy0) [79]. All the tools were used with default parameters.

FASTA files were also introduced in Geneious bioinformatics software version 2024.0 (http://www.geneious.com; accessed on 1 February 2024), which was used to obtain the number of sequences, the percentage of GCs, and the sequence length. The sequencing coverage *c* describes the average number of reads that align to known bases; at higher levels of coverage, each base is covered by a greater number of aligned sequence reads. To estimate and achieve the desired sequencing coverage level, the Lander/Waterman equation [80,81,82] was used, that is, *c* = (*L × N*)/*G*, where *c* is the coverage, *L × N* is the number of bases sequenced (*L* is the read length in bp and *N* is the number of reads), and *G* is the haploid genome length in bp.

#### 4.5.2. Polishing and Annotation

The tool “medaka consensus pipeline—Assembly polishing via neural networks” (Galaxy Version 1.7.2+galaxy0) [83] was used to polish the final assembly obtained from Raven tool FASTA files. The obtained draft genome was used for subsequent analysis. The “Prokka—Prokaryotic genome annotation” (Galaxy Version 1.14.6+galaxy1) tool [84,85] was used for genome annotation. Both tools were used with default parameters.

#### 4.5.3. Typing, Antimicrobial Resistance and Virulence

The tool “ABRicate—Mass screening of contigs for antimicrobial and virulence genes” (Galaxy Version 1.0.1) [86] was used to identify the presence of antimicrobial resistance genes. The Center for Genomic Epidemiology (CGE; http://www.genomicepidemiology.org/; accessed on 1 March 2024) is entirely noncommercial and operates several free online bioinformatics services and tools. MLST 2.0.9 [87] and PlasmidFinder 2.0.1 [88] tools were used for multilocus sequence typing (MLST) and identification of plasmids, respectively. Four prerequisites have been considered for their selection for MLST: (i) the alignment length (also known as the high-scoring segment pair or HSP) must equal the allele length; (ii) the identity must be 100%; (iii) the coverage must be 100%; and (iv) the number of gaps in the HSP must be zero. The length of the alignment between the best-matching MLST allele in the database and the corresponding sequence in the genome of the *S. uberis* strains studied is referred to as the alignment length. The length of the best-matching MLST allele in the database is called the allele length. The percentage of identity is the percentage of nucleotides that are identical in the best-matching MLST allele in the database and the corresponding sequence in the genome of *S. uberis* strains.

The Cano-wgMLST_BacCompare platform (https://github.com/cenesis/cano-wgMLST; accessed on 1 March 2024) was used for epidemiological investigations and comparative genomic analysis [89]. The Virulence Factor Database (VFDB; http://www.mgc.ac.cn/VFs/main.htm; accessed on 1 March 2024) was used for the prediction of virulence factors (VFs) and related genes for each *S. uberis* strain [90].

The FASTA files of the isolates were used with CGE’s PathogenFinder 1.1 [91] tool for predicting the pathogen action of the studied strains toward human cells.

## 5. Conclusions

This study aimed to identify the genetic differences and similarities among *S. uberis* strains previously isolated from bovine mastitis in northwestern Spain, a region ranked among the top ten European milk producers. Current knowledge of the genomic characteristics of this bacterium is limited, underscoring the need for further research.

Different gene expression patterns allow *S. uberis* to adapt to different environmental conditions and stress situations. Understanding these adaptations is essential for the design of public health control strategies. Our study revealed a great diversity of allelic profiles or STs, indicating that the spread of *S. uberis* in NW Spain is more heterogeneous than contagious. On the basis of graphical analysis, no correlations were found between the known sequencing types of this study and the geographical characteristics of the area.

The findings highlight that *S. uberis* exhibits diverse gene expression patterns, allowing adaptation to a range of environmental conditions and stress factors. Understanding these adaptations is essential for developing effective public health control strategies. Our results revealed a high diversity of allelic profiles and sequence types, suggesting that the spread of *S. uberis* in northwestern Spain is more heterogeneous than clonal. Graphical analysis showed no correlation between the identified sequence types and the geographical characteristics of the sampling areas.

The isolates analyzed in this study showed considerable intraspecies variability and global diversity, consistent with observations in other regions. The relatively large proportion of accessory genes detected indicates an increased adaptive capacity, particularly to the challenging udder environment, which harbors diverse microbial communities competing for resources.

Of particular concern is the role of *S. uberis* as a potential reservoir of antimicrobial resistance genes, which may be transmitted to other bacteria, pathogens, and commensals. Monitoring the antimicrobial susceptibility of *S. uberis* is crucial for guiding veterinary treatment of bovine mastitis and for supporting the One Health approach. The antimicrobial resistance detected in several strains raises concerns regarding the effectiveness of current treatment strategies in dairy farming.

Although *S. uberis* is rarely associated with human infections, reported cases do exist. Importantly, all the strains analyzed in this study were predicted to have pathogenic potential in humans, emphasizing the relevance of continued surveillance and characterization of this bacterium.

## Figures and Tables

**Table 1:** Phenotypic antimicrobial profile and genomic resistance to antibiotics from the *S. uberis* strains studied. The number of virulence factors (VF) has been included. TET, tetracycline; ERY, erythromycin; CLI, clindamycin; CRO, ceftriaxone.

Strains Code	Phenotypic Antimicrobial Profile	Resistance Genes	Number of VF
1121090	TET, ERY, CLI	*ant(6)-Ia_2; lnu(B)_2; lsa(E)_1*	8
1121094	-	*erm(B)_18*	11
1121108	-	*lnu(C)_1*	10
1121118	-	*lnu(C)_1*	11
1121191		*lnu(C)_1*	13
1121208		*mph(B)_1*	11
1121227		*tet(L)_2; tet(M)_5; ant(6)-Ia_2; lnu(B)_2; lsa(E)_1*	14
1121287	-	*erm(B)_18*	-
1121292	TET, CLI	*lsa(E)_1; lnu(B)_2; ant(6)-Ia_2; tet(L)_2; tet(M)_5*	13
1121295	-	*erm(B)_18; lnu(C)_1*	10
1121300	-	-	12
1121323	TET, CLI	*lsa(E)_1; lnu(B)_2; ant(6)-Ia_2; tet(M)_5; tet(L)_2*	11
1121338	-	-	10
1121346		*erm(B)_18;* *aph(3′)-III_1;* *ant(6)-Ia_1;* *tet(O)_3*	-
1121350		*lnu(C)_1;* *erm(B)_18;* *aph(3′)-III_1;* *ant(6)-Ia_1;* *tet(O)_3*	11
1121751	CLI	*lsa(E)_1; lnu(B)_2; ant(6)-Ia_2*	6
1121757	TET, CLI	*lsa(E)_1; lnu(B)_2; ant(6)-Ia_2; mph(B)_1; tet(M)_5; tet(L)_2*	13
1121772		*tet(L)_2; tet(M)_5; ant(6)-Ia_2; lnu(B)_2; lsa(E)_1; lnu(C)_1*	10
1121774		*lsa(E)_1;lnu(B)_2;ant(6)-Ia_2*	-
1121776		*lnu(B)_2; lsa(E)_1; ant(6)-Ia_2; erm(B)_18*	11
1121974		*tet(O)_3;* *erm(B)_18;* *ant(6)-Ia_3*	13
1121980	TET, ERY, CLI	*tet(O)_3;* *ant(6)-Ia_1;* *aph(3′)-III_1;* *erm(B)_18*	13
1121981		-	10
1122022		*erm(B)_18; aph(3′)-III_1; ant(6)-Ia_1; lnu(B)_2; lsa(E)_1*	9
1122039		*ant(6)-Ia_2; lnu(B)_2; lsa(E)_1; tet(S)_3*	11
1122285	TET, ERY, CLI	*lsa(E)_1; lnu(B)_2; ant(6)-Ia_2; erm(B)_18; aph(3′)-III_1; ant(6)-Ia_1; tet(O)_3*	9
1122348	TET, ERY, CLI	*ant(6)-Ia_3;* *erm(B)_18;* *tet(O)_3;* *tet(L)_2;* *tet(M)_5;*	10
1122419		*ant(6)-Ia_2; lnu(B)_2; lsa(E)_1*	10
1122603	TET, CLI	*tet(L)_2; tet(M)_5; ant(6)-Ia_2; lnu(B)_2; lsa(E)_1*	14
1122648	ERY, CLI	*erm(B)_18*	8
1122846	-	-	9
1122847	CLI	*lsa(E)_1; lnu(B)_2; ant(6)-Ia_2*	11
1122852	TET, CLI, CRO	*lsa(E)_1; lnu(B)_2; ant(6)-Ia_2; tet(L)_2; tet(M)_5; lnu(C)_1*	9
1122911		*tet(L)_2;* *tet(M)_5; ant(6)-Ia_2; lnu(B)_2;* *lsa(E)_1;* *lnu(D)_1;* *mph(B)_1*	10
1122931		*tet(M)_5; lsa(E)_1; lnu(B)_2; ant(6)-Ia_2*	10
1122956		*erm(B)_18;* *lnu(D)_1*	10

**Table 2:** Resistance gene classification according to their frequency of appearance and type (left side of the table). Virulence factors (VF) major classes from each strain and their relationship to their related genes (right side of the table).

Strains	*lnu*	*ant(6)-Ia*	*lsa(E)_1*	*tet*	*erm(B)_18*	*aph(3′)-III_1*	*mph(B)_1*	Strains	I	II	III	IV	V	VI	VII	VIII	IX	X	XI	XII	XIII	XIV	XV	XVI	XVII	XVIII	XIX
1121090	B	2	+					1121090																			
1121094					+			1121094																			
1121108	C							1121108																			
1121118	C							1121118																			
1121191	C							1121191																			
1121208							+	1121208																			
1121227	B	2	+	M, L				1121227																			
1121287					+			1121287																			
1121292	B	2	+	M, L				1121292																			
1121295	C				+			1121295																			
1121300								1121300																			
1121323	B	2	+	M, L				1121323																			
1121338								1121338																			
1121346		1		O	+	+		1121346																			
1121350	C	1		O	+	+		1121350																			
1121751	B	2	+					1121751																			
1121757	B	2	+	M, L			+	1121757																			
1121772	B, C	2	+	M, L				1121772																			
1121774	B	2	+					1121774																			
1121776	B	2	+		+			1121776																			
1121974		3		O	+			1121974																			
1121980		1		O	+	+		1121980																			
1121981								1121981																			
1122022	B	1	+		+	+		1122022																			
1122039	B	2	+	S				1122039																			
1122285	B	2, 1	+	O	+	+		1122285																			
1122348		3		M, L, O	+			1122348																			
1122419	B	2	+					1122419																			
1122603	B	2	+	M, L				1122603																			
1122648					+			1122648																			
1122846								1122846																			
1122847	B	2	+					1122847																			
1122852	B, C	2	+	M, L				1122852																			
1122911	B, D	2	+	M, L			+	1122911																			
1122931	B	2	+	M				1122931																			
1122956	D				+			1122956																			

Types of genes ordered from most to least frequent: Gene *lnu*: B, gene *lnu(B)_2* (18/36 strains); C, gene *lnu(C)_1* (7/36); D, gene *lnu(D)_1* (2/36). Gene *ant(6)-Ia*: “2”, gene *ant(6)-Ia*_2 (17/36); “1”, gene *ant(6)-Ia_1* (4/36); “3”, gene *ant(6)-Ia_3* (2/36). Gene *lsa(E)_1* (18/36). Gene *tet*: M, gene *tet(M)_5* (10/36); L, *tet(L)_2* (9/36); O, *tet(O)_3* (6/36); S, *tet(S)_3* (1/36). Gene *erm(B)_18* (13/36). Gene *aph(3′)-III_1* (5/36). Gene *mph(B)_1* (3/36). Virulence factors (VFs): I: Agglutinin receptor. II: Fibronectin-binding proteins. III: Laminin-binding proteins. IV: Streptococcal lipoprotein rotamase A. V: Streptococcal plasmid receptor/GAPDH. VI: rlrA islet. VII: Hyaluronidase. VIII: Streptococcal enolase. IX: Capsule. X: polysaccharide capsule (Bacillus). XI: Exopolysaccharide (Haemophilus). XII: Rib. XIII: Pneumococcal surface antigen A/Metal binding protein SloC. XIV: C3-degrading protease. XV: C5a peptidase. XVI: Serine protease. XVII: Trigger factor. XVIII: Trehalose-recycling ABC transporter (Mycobacterium). XIX: LPS (Francisella).

**Table 3:** Number and percentage of strains with detected virulence factors (VF).

VF Class	VF	Related Genes	Strains (%) ^1^
Adherence	Agglutinin receptor	Undetermined	5 (15.15)
	Fibronectin-binding proteins	*fbp54*	33 (100)
	Laminin-binding proteins	*lmb*	30 (90.91)
	Streptococcal lipoprotein rotamase A	*-/slrA*	13 (39.39)
	Streptococcal plasmid receptor/GAPDH	*plr/gapA*	21 (63.64)
	rlrA islet	*srtC*	1 (3.03)
Enzyme	Hyaluronidase	*hyIB*	12 (36.36)
	Streptococcal enolase	*eno*	31 (93.94)
Immune evasion	Capsule	Undetermined	33 (100)
	Polysaccharide capsule (*Bacillus*)	*/galE*	11 (33.33)
	Exopolysaccharide (*Haemophilus*)	*galE*	2 (6.06)
Immunoreactive antigen	Rib	*rib*	4 (12.12)
Manganese uptake	Pneumococcal surface antigen A/Metal binding protein SloC	*psaA*	33 (100)
Protease	C3-degrading protease	*cppA*	33 (100)
	C5a peptidase	*scpA/scpB*	24 (72.73)
	Serine protease	*htrA/degP*	33 (100)
	Trigger factor	*tig/ropA*	21 (63.64)
Cell surface components	Trehalose-recycling ABC transporter (*Mycobacterium*)	*sugC*	10 (30.30)
Serum resistance and immune evasion	LPS (*Francisella*)	*wbtP*	2 (6.06)

^1^ From a total of 33 strains.

## Data Availability

The raw data supporting the conclusions of this article were deposited in the NCBI GenBank upon the Bioproject PRJNA1321121.

## Supplementary Materials

The following supporting information can be downloaded at: https://www.mdpi.com/article/10.3390/antibiotics14111059/s1, Supplementary File S1: Sequencing statistics obtained from the Nanoplot tool and/or Geneious software; Supplementary File S2A: MLST loci against which the input sequence has been aligned; Supplementary File S2B: Sequence types (STs) and nearest STs that the MLST server associated with the submitted data; Supplementary File S2C: Most discriminatory loci (416 with ˃95% strain occurrence) from wgMLST; Supplementary File S2D: Venn diagram output with ellipses from wgMLST (36 *S. uberis* strains plus 2 reference strains); Supplementary File S3A: Venn diagram output with ellipses from wgMLST (36 *S. uberis* strains without reference strains); Supplementary File S3B: Genetic relatedness tree from wgMLST; Supplementary File S3C: Most discriminatory loci (402 with ˃95% strain occurrence) from wgMLST; Supplementary File S3D: Genetic relatedness tree from wgMLST (36 strains); Supplementary File S4: Predicted resistance phenotype to antibiotics from the *S. uberis* strains studied; Supplementary File S5A: Plasmids results from PlasmidFinder tool; Supplementary File S5B: Additional information related to the virulence factors that contribute to the pathogenicity of *S. uberis*. Different open reading frames (orf) are shown for each gene; Supplementary File S5C: Prediction of possible human pathogenicity using PathogenFinder tool.
