# Supplementary material for: Genomic Characterization and Antimicrobial Resistance Profile of Streptococcus uberis Strains Isolated from Cows with Mastitis from Northwestern Spain
Source: Antibiotics (Basel). 2025 Oct 23;14(11):1059. doi: 10.3390/antibiotics14111059 (PMC12649216; doi:10.3390/antibiotics14111059)
Supplement: Supplementary file 1 [file antibiotics-14-01059-s001.zip › antibiotics-3895307-supplementary/Supplementary File S1.pdf]

Supplementary File S1. Sequencing statistics obtained from the Nanoplot tool and/or Geneious software.

Isolates	Nanoplot tool[1]							Geneious software[2]			
	N	bp	M	L	SDL	n50	lrQ1	# Seq	% GC	Seq L	C
1121090	96160	88981468	438	925,3	2236,8	1742	54860	2	36.7	1968763	45,20
1121094	131945	109197931	524	827,6	1181,7	1146	74743	7	36.6	1890584	57.76
1121108	104538	358189305	960	3426,4	5739,8	10106	80502	1	36.5	2005438	178,61
1121118	310842	857533830	737	2758,7	5176,7	9696	124526	8	36.5	2165966	395,91
1121191	106899	107377843	474	1004,5	1897,6	2081	54069	2	36.6	2055940	52,23
1121208	206687	1117849110	2977	5408,4	6726,4	11072	89778	1	36.3	1905405	586,67
1121227	113648	111442148	352	980,6	2029,7	3656	91811	3	36.5	1967701	56,64
1121287	138682	264859771	566	1909,8	3890,5	6726	73592	7	36.5	2274136	116,47
1121292	276554	213828666	409	773,2	1407,1	1354	48656	1	36.5	2061008	103,75
1121295	216331	964388185	2197	4457,9	5778,6	9527	132478	3	36.6	1981311	486,74
1121300	79897	78610530	491	983,9	1820,3	1958	44852	3	36.5	1950420	40,30
1121323	106936	114628733	554	1071,9	1988,1	1854	49988	2	36.8	1996765	57,41
1121338	88016	117750015	561	1337,8	2601,5	3247	37296	4	36.4	1932930	60,92
1121346	98272	184716480	914	1879,6	3333,2	3692	87395	1	36.6	1993231	92,67
1121350	69629	71392431	393	1025,3	2555,4	2868	64850	20	36.4	2007551	35,56
1121751	79551	189534072	668	2382,5	4554,5	8246	66437	2	36.4	2036258	93,08
1121757	82857	346430918	1040	4181,1	6746,5	12525	74488	1	36.6	1920311	180,40
1121772	258681	224084036	474	866,3	1669,2	1310	59077	2	36.6	2133519	105,03
1121774	339998	283435678	467	833,6	1338,3	1266	43857	3	36.4	1985960	142,72
1121776	507352	390292685	485	769,3	948,1	1080	61717	4	36.4	2019945	193,22
1121974	263264	330277100	585	1254,5	2186,3	2648	92806	3	36.7	2052042	160,95
1121980	312752	460505850	658	1472,4	2726,7	3245	70756	3	36.5	2063052	223,22
1121981	83186	163129880	1230	1961	2231,9	3265	56354	2	36.5	2011996	81,08
1122022	191621	163403963	553	852,7	1174	1160	45224	1	36.5	2014907	81,10
1122039	210307	200938891	471	955,5	1768,7	1723	61138	3	36.7	1945041	103,31
1122285	152933	241839042	491	1581,3	3320,9	5392	64724	1	36.4	2135116	113,27
1122348	83329	352994100	1641	4236,1	6074,5	10431	77914	2	36.6	2137342	165,16
1122419	173731	181409963	577	1044,2	1615,2	1725	57995	2	36.5	1900040	95,48
1122603	125917	250529664	903	1989,6	2914,9	4464	50473	1	36.7	1881690	133,14
1122648	280066	1223284361	1975	4367,8	6299,6	9031	263580	2	36.6	1988991	615,03
1122846	401889	2533555455	3703	6304,1	7253,7	12191	88471	1	36.6	1939819	1306,08
1122847	99719	169717982	579	1702	2946,1	4975	50712	1	36.5	1948507	87,10
1122852	103851	88633490	577	853,5	1062,3	1176	43077	7	36.6	1958205	45,26
1122911	98051	154647989	439	1577,2	3798,6	7259	61899	1	36.4	2050223	75,43
1122931	61044	379219601	3742,5	6212,2	7179	12331	102180	1	36.7	1914931	198,03
1122956	76152	73255006	457	962	1815,9	1851	47684	5	36.8	1942277	37,72

[1] Number of reads (N); number of bases (bp); median read length (M); mean read length (L); standard deviation of read length (SDL). [2] The shortest contig length that needs to be included for covering 50% of the genome (n50); the longest reads with a Q score of 1, meaning a 10% error rate (lrQ1); number of sequences (# Seq); percentage of guanine and cytosine in a sequence (GC content: %GC); sequence length (Seq L); C (coverage).
