# Supplementary material for: Genomic Characterization and Antimicrobial Resistance Profile of Streptococcus uberis Strains Isolated from Cows with Mastitis from Northwestern Spain
Source: Antibiotics (Basel). 2025 Oct 23;14(11):1059. doi: 10.3390/antibiotics14111059 (PMC12649216; doi:10.3390/antibiotics14111059)

Supplementary File S2.A. MLST loci against which the input sequence has been aligned. Best matching MLST alleles in the database for each locus (e.g., arcC_1).

Strain	Loci[a]							Seq
	arcC	ddl	gki	recP	tdk	tpi	yqiL	Type
1121090	-	ddl_1	-	-	tdk_3	-	-	U
1121094	arcC_1	_1	-	recP_1	_1	tpi_1	yqiL_3	U
1121108	_35	_56	gki_24	_2	_17	_1	-	U
1121118	_35	_56	_24	_2	_17	_1	-	U
1121191[c]	_1	_1	_7	_2	_65	_1	_10	U
1121208[c]	_1	_1	_2	_1	_2	_1	_3	6
1121227[c]	_1	_1	_4	_1	_2	_1	_3	307
1121287	_21![b]	_16	_4	_27	-	_13	-	U
1121292	_1	-	_4	_1	_2	_1	-	U
1121295[c]	_1	_49	_2	_2	_7	_3	_7	U
1121300[c]	_1	_1	_5	_1	_17	_1	_6	U
1121323	_1	_1	_4	_1	_76	-	_3	U
1121338[c]	_1	_1	_2	_2	_17	_1	_3	184
1121346	-	_42	-	-	_2	-	-	U
1121350	_3	_4	_4	_4	-	_2	_3	U
1121751	-	_2	-	-	_1	-	-	386?*
1121757[c]	_2	_1	_10	_1	_2	_1	_3	U
1121772	-	-	-	-	_2	-	-	U
1121774	_56	_1	_3	_2![b]	_2	_3	_3	815!
1121776	_9	_1	_5	_1	_2	_11	-	U
1121974	-	_23	-	_2	_2	_3	_10	U
1121980	-	_1	-	-	_65	-	_7	U
1121981	-	_1	-	-	_2	-	-	U
1122022	-	_1	-	-	_11	-	-	U
1122039	-	_3	_65	-	_65	_1	_3	U
1122285	-	_1	-	-	_49	-	-	U
1122348	-	_1	-	-	_46	-	-	U
1122419	-	_1	-	-	_65	-	-	U
1122603[c]	_1	_1	_43	_1	_2	_1	_3	U
1122648	-	_10	-	-	_3	-	-	U
1122846	-	_1	-	-	_13	-	-	U
1122847[c]	_1	_1	_4	_2	_122	_1	_3	U
1122852	-	_1	-	-	_2	-	-	U
1122911	-	-	-	-	_2	-	-	U
1122931[c]	_1	_1	_4	_1	_2	_1	_3	307
1122956	_2	_42	_5	_2	-	_1	_3	U

[a] The alignment length and the allele length for each locus are equal for the strains studied: arcC (carbamate kinase; 419 bp), ddl (D-alanine-D-alanine ligase; 357), gki (glucokinase; 454), recP (transketolase; 372), tdk (thymidine kinase; 500), tpi (triosephosphate isomerase; 373), and yqiL (acetyl-CoA acetytransferase; l439). [b] Multiple alleles in the database of the MLST tool perfectly match the corresponding sequence in the genome of the S. uberis strain. [c] Ten out of 36 strains had alleles for all the loci. U: Unknown. ?* Alleles with less than 100% identity and 100% coverage found. ! When alleles with multiple perfect fits are found, multiple STs might be found.

Supplementary File S2.B. Sequence types (STs) and nearest STs that the MLST server associated with the submitted data.

Strain	ST	Nearest ST
1121090	U	793
1121094	U	1436, 1437
1121108	U	1339, 551, 709, 161, 184, 216, 243, 765, 150, 749, 91, 671, 140, 642, 991, 142, 1191, 712, 711, 123, 112, 118, 410, 252, 144, 673, 162, 141, 285, 145, 668, 829, 840, 834
1121118	U	829, 216, 1339, 671, 243, 142, 150, 112, 144, 673, 91, 123, 1191, 141, 551, 410, 252, 118, 834, 140, 749, 161, 642, 285, 668, 991, 711, 162, 145, 709, 840, 765, 184, 712
1121191	U	955, 693, 64, 932, 613, 799, 694, 647, 11
1121208	6	-
1121227	307	-
1121287	U	212, 742, 138, 1206, 1144, 449, 570, 196
1121292	U	307
1121295	U	1370
1121300	U	741, 745
1121323	U	421, 878, 307, 876, 1141, 1437
1121338	184	-
1121346	U	1408, 1403, 895, 1343, 812, 1331
1121350	U	1303
1121751	386?*	-
1121757	U	982, 1216, 316, 1065, 361, 1213
1121772	U	307, 1149, 510, 77, 63, 4, 329, 1147, 13, 1325, 1390, 572, 5, 7, 2, 944, 948, 332, 6, 353
1121774	815!	-
1121776	U	959, 1421, 1280, 1325, 986, 1146, 490, 589, 1270, 248, 1315, 633, 63, 356, 1065, 948, 782, 479, 353, 1273, 1335
1121974	U	905, 930, 981, 479, 1139
1121980	U	1114, 1146, 1127
1121981	U	454, 1283, 794, 901, 14, 990, 696, 919, 924
1122022	U	367, 1129, 793, 338
1122039	U	399, 876, 70, 532
1122285	U	923, 990, 1283, 901, 304, 879
1122348	U	1367
1122419	U	1157
1122603	U	1440, 77, 1216, 63, 307, 332, 13, 1348, 6
1122648	U	323, 1317, 322, 27
1122846	U	534, 1406, 267, 637
1122847	U	1438, 551, 1377, 501, 1095, 906
1122852	U	361, 1213, 1216, 982, 1065, 316
1122911	U	929, 1146, 1137, 782, 1325
1122931	307	-
1122956	U	880

U, unknown

?* Alleles with less than 100% identity and 100% coverage.

! Alleles with multiple perfect fits found; multiple STs might be found.

Supplementary File S2.C. The most discriminatory loci (416 with >95% of strains occurrence) from wgMLST. The 101 highly discriminatory loci are also shown (in bold). Thirty-six plus two reference strains.

#	Locus	Gene name	Occurrence (%)	Annotation
1	SAL0000001	*pepF1_1*	94.7	Oligoendopeptidase F, plasmid
2	SAL0000002	*mutS2*	94.7	Endonuclease MutS2
3	SAL0000003	*rpmH*	94.7	50S ribosomal protein L34
**4**	**SAL0000004**	***asnA***	**94.7**	**Aspartate--ammonia ligase**
5	SAL0000005	*group_14592*	94.7	Putative phosphatase
6	SAL0000006	*group_1526*	94.7	hypothetical protein
7	SAL0000007	*group_1594*	94.7	Putative acetyltransferase
8	SAL0000008	*clpX*	94.7	hypothetical protein
**9**	**SAL0000009**	***group_1919***	**94.7**	**hypothetical protein**
**10**	**SAL0000010**	***rplL***	**94.7**	**50S ribosomal protein L7/L12**
11	SAL0000011	*gluP*	94.7	Rhomboid protease GluP
12	SAL0000012	*rplO*	94.7	50S ribosomal protein L15
**13**	**SAL0000013**	***rpmC***	**94.7**	**50S ribosomal protein L29**
14	SAL0000014	*group_3282*	94.7	hypothetical protein
15	SAL0000015	*group_3297*	94.7	pyridoxal phosphate (PLP) phosphatase
16	SAL0000016	*aguA*	94.7	Putative agmatine deiminase
17	SAL0000017	*group_3530*	94.7	hypothetical protein
18	SAL0000018	*azr_2*	94.7	NADPH azoreductase
19	SAL0000019	*group_3966*	94.7	hypothetical protein
20	SAL0000020	*group_4041*	94.7	hypothetical protein
21	SAL0000021	*group_4061*	94.7	hypothetical protein
22	SAL0000022	*group_4145*	94.7	hypothetical protein
23	SAL0000023	*adhR*	94.7	HTH-type transcriptional regulator AdhR
24	SAL0000024	*group_4173*	94.7	hypothetical protein
25	SAL0000025	*group_4220*	94.7	hypothetical protein
26	SAL0000026	*mscL*	94.7	Large-conductance mechanosensitive channel
27	SAL0000027	*ftsX*	94.7	Cell division protein FtsX
28	SAL0000028	*hprK*	94.7	HPr kinase/phosphorylase
29	SAL0000029	*rpsT*	94.7	30S ribosomal protein S20
**30**	**SAL0000030**	***group_4414***	**94.7**	**Ribosome-associated factor Y**
31	SAL0000031	*group_4444*	94.7	hypothetical protein
32	SAL0000032	*group_4452*	94.7	hypothetical protein
33	SAL0000033	*moeZ_2*	94.7	putative adenylyltransferase/sulfurtransferase MoeZ
34	SAL0000034	*polC_1*	94.7	DNA polymerase III PolC-type
35	SAL0000035	*ybaN*	94.7	Inner membrane protein YbaN
36	SAL0000036	*spxA_1*	94.7	Regulatory protein Spx
37	SAL0000037	*nudC*	94.7	NADH pyrophosphatase
38	SAL0000038	*pgsA*	94.7	CDP-diacylglycerol--glycerol-3-phosphate 3-phosphatidyltransferase
39	SAL0000039	*deoC1*	94.7	Deoxyribose-phosphate aldolase 1
40	SAL0000040	*group_4587*	94.7	hypothetical protein
41	SAL0000041	*yvoA_2*	94.7	HTH-type transcriptional repressor YvoA
42	SAL0000042	*glgA*	94.7	Glycogen synthase
43	SAL0000043	*glnM*	94.7	putative glutamine ABC transporter permease protein GlnM
44	SAL0000044	*msrA*	94.7	Peptide methionine sulfoxide reductase MsrA
45	SAL0000045	*yeeN*	94.7	putative transcriptional regulatory protein YeeN
46	SAL0000046	*trxC*	94.7	Thioredoxin-2
47	SAL0000047	*dexB*	94.7	Glucan 1,6-alpha-glucosidase
48	SAL0000048	*mapP*	94.7	Maltose 6'-phosphate phosphatase
**49**	**SAL0000049**	***group_4846***	**94.7**	**hypothetical protein**
50	SAL0000050	*group_4883*	94.7	hypothetical protein
51	SAL0000051	*tadA_1*	94.7	tRNA-specific adenosine deaminase
**52**	**SAL0000052**	***group_4909***	**94.7**	**Nucleoid-associated protein**
53	SAL0000053	*group_4924*	94.7	hypothetical protein
54	SAL0000054	*group_4925*	94.7	hypothetical protein
55	SAL0000055	*skc*	94.7	Streptokinase C precursor
56	SAL0000056	*pat*	94.7	Phosphinothricin N-acetyltransferase

57	SAL0000057	*lptB_1*	94.7	Lipopolysaccharide export system ATP-binding protein LptB
58	SAL0000058	*ddl*	94.7	D-alanine--D-alanine ligase
**59**	**SAL0000059**	***yniC***	**94.7**	**Phosphorylated carbohydrates phosphatase**
**60**	**SAL0000060**	***group_4991***	**94.7**	**hypothetical protein**
61	SAL0000061	*mtrR*	94.7	HTH-type transcriptional regulator MtrR
62	SAL0000062	*group_5034*	94.7	FtsX-like permease family protein
**63**	**SAL0000063**	***gph***	**94.7**	**Phosphoglycolate phosphatase**
**64**	**SAL0000064**	***czcO***	**94.7**	**putative oxidoreductase CzcO**
65	SAL0000065	*mutM*	94.7	Formamidopyrimidine-DNA glycosylase
66	SAL0000066	*feuB*	94.7	Iron-uptake system permease protein FeuB
67	SAL0000067	*group_5071*	94.7	hypothetical protein
68	SAL0000068	*trmL*	94.7	Putative tRNA (cytidine(34)-2'-O)-methyltransferase
**69**	**SAL0000069**	***rpsM***	**94.7**	**30S ribosomal protein S13**
70	SAL0000070	*group_5271*	94.7	preprotein translocase subunit YajC
**71**	**SAL0000071**	***prmA***	**94.7**	**Ribosomal protein L11 methyltransferase**
72	SAL0000072	*group_5282*	94.7	Single-stranded DNA-binding protein ssb
73	SAL0000073	*group_5289*	94.7	preprotein translocase subunit SecY
74	SAL0000074	*purC*	94.7	Phosphoribosylaminoimidazole-succinocarboxamide synthase
**75**	**SAL0000075**	***mreC***	**94.7**	**Cell shape-determining protein MreC precursor**
76	SAL0000076	*recU*	94.7	Holliday junction resolvase RecU
77	SAL0000077	*glyQ*	94.7	Glycine--tRNA ligase alpha subunit
78	SAL0000078	*group_5310*	94.7	copper exporting ATPase
**79**	**SAL0000079**	***gmuF***	**94.7**	**putative mannose-6-phosphate isomerase GmuF**
**80**	**SAL0000080**	***group_5325***	**94.7**	**hypothetical protein**
81	SAL0000081	*trxA*	94.7	Thioredoxin
82	SAL0000082	*rmuC*	94.7	RmuC family protein
**83**	**SAL0000083**	***thiN***	**94.7**	**Thiamine pyrophosphokinase**
84	SAL0000084	*murR_2*	94.7	HTH-type transcriptional regulator MurR
85	SAL0000085	*dut*	94.7	Deoxyuridine 5'-triphosphate nucleotidohydrolase
86	SAL0000086	*azr_1*	94.7	NADPH azoreductase
87	SAL0000087	*group_5367*	94.7	hypothetical protein
**88**	**SAL0000088**	***yrrK***	**94.7**	**Putative Holliday junction resolvase**
89	SAL0000089	*smc_2*	94.7	Chromosome partition protein Smc
90	SAL0000090	*group_5382*	94.7	hypothetical protein
91	SAL0000091	*group_5389*	94.7	5'-nucleotidase
92	SAL0000092	*spo0C*	94.7	Chromosome-partitioning protein Spo0J
93	SAL0000093	*tlyA*	94.7	Hemolysin A
94	SAL0000094	*focA*	94.7	putative formate transporter 1
95	SAL0000095	*naiP*	94.7	Putative niacin/nicotinamide transporter NaiP
96	SAL0000096	*group_5426*	94.7	Cupin domain protein
97	SAL0000097	*group_5433*	94.7	hypothetical protein
**98**	**SAL0000098**	***group_5438***	**94.7**	**hypothetical protein**
99	SAL0000099	*group_5439*	94.7	hypothetical protein
**100**	**SAL0000100**	***coaBC_1***	**94.7**	**Coenzyme A biosynthesis bifunctional protein CoaBC**
101	SAL0000101	*xerD*	94.7	Tyrosine recombinase XerD
102	SAL0000102	*group_5472*	94.7	hypothetical protein
103	SAL0000103	*vioD*	94.7	Capreomycidine synthase
104	SAL0000104	*endA*	94.7	DNA-entry nuclease
105	SAL0000105	*group_5514*	94.7	hypothetical protein
106	SAL0000106	*group_5527*	94.7	hypothetical protein
**107**	**SAL0000107**	***sdpI***	**94.7**	**Immunity protein SdpI**
108	SAL0000108	*group_5550*	94.7	hypothetical protein
109	SAL0000109	*tmk*	94.7	Thymidylate kinase
110	SAL0000110	*ytgP_2*	94.7	putative cell division protein YtgP
111	SAL0000111	*group_5561*	94.7	putative acetyltransferase
112	SAL0000112	*tcyA*	94.7	L-cystine-binding protein TcyA precursor
113	SAL0000113	*group_5566*	94.7	Phosphorylated carbohydrates phosphatase
114	SAL0000114	*group_5575*	94.7	Deoxyguanosine kinase
115	SAL0000115	*metQ*	94.7	D-methionine-binding lipoprotein MetQ precursor
116	SAL0000116	*yumC*	94.7	Ferredoxin--NADP reductase 2
117	SAL0000117	*group_5595*	94.7	Helix-turn-helix
118	SAL0000118	*rlmCD_1*	94.7	23S rRNA (uracil-C(5))-methyltransferase RlmCD
119	SAL0000119	*group_5673*	94.7	Flavodoxin
120	SAL0000120	*ywpJ*	94.7	Putative phosphatase YwpJ

121	SAL0000121	*mhqR*	94.7	HTH-type transcriptional regulator MhqR
122	SAL0000122	*group_5718*	94.7	hypothetical protein
**123**	**SAL0000123**	***group_5719***	**94.7**	**hypothetical protein**
124	SAL0000124	*group_5724*	94.7	hypothetical protein
125	SAL0000125	*murR_1*	94.7	HTH-type transcriptional regulator MurR
126	SAL0000126	*group_5737*	94.7	hypothetical protein
127	SAL0000127	*rplF*	94.7	50S ribosomal protein L6
128	SAL0000128	*rplN*	94.7	50S ribosomal protein L14
**129**	**SAL0000129**	***rpsC***	**94.7**	**30S ribosomal protein S3**
130	SAL0000130	*rplW*	94.7	50S ribosomal protein L23
**131**	**SAL0000131**	***group_5753***	**94.7**	**DNA utilization protein GntX**
132	SAL0000132	*rsmB*	94.7	Ribosomal RNA small subunit methyltransferase B
133	SAL0000133	*luxS*	94.7	S-ribosylhomocysteine lyase
134	SAL0000134	*group_5759*	94.7	hypothetical protein
135	SAL0000135	*group_5774*	94.7	Bacterial ABC transporter protein EcsB
136	SAL0000136	*group_5778*	94.7	Acyltransferase family protein
**137**	**SAL0000137**	***lytG***	**94.7**	**Exo-glucosaminidase LytG precursor**
138	SAL0000138	*ccpN*	94.7	Transcriptional repressor CcpN
**139**	**SAL0000139**	***group_5790***	**94.7**	**hypothetical protein**
140	SAL0000140	*bglA_1*	94.7	Aryl-phospho-beta-D-glucosidase BglA
**141**	**SAL0000141**	***rpsN2***	**94.7**	**Alternate 30S ribosomal protein S14**
142	SAL0000142	*tsaB*	94.7	tRNA threonylcarbamoyladenosine biosynthesis protein TsaB
143	SAL0000143	*purR*	94.7	Pur operon repressor
144	SAL0000144	*gtaB*	94.7	UTP--glucose-1-phosphate uridylyltransferase
145	SAL0000145	*group_5803*	94.7	PTS system glucitol/sorbitol-specific transporter subunit IIA
146	SAL0000146	*licR_2*	94.7	putative licABCH operon regulator
147	SAL0000147	*ulaC_1*	94.7	Ascorbate-specific phosphotransferase enzyme IIA component
148	SAL0000148	*rluC*	94.7	Ribosomal large subunit pseudouridine synthase C
149	SAL0000149	*ruvA*	94.7	Holliday junction ATP-dependent DNA helicase RuvA
**150**	**SAL0000150**	***sdhB***	**94.7**	**L-serine dehydratase, beta chain**
151	SAL0000151	*glcU_2*	94.7	putative glucose uptake protein GlcU
152	SAL0000152	*tilS*	94.7	tRNA(Ile)-lysidine synthase
153	SAL0000153	*group_5859*	94.7	molybdopterin biosynthesis protein MoeB
**154**	**SAL0000154**	***murG***	**94.7**	**UDP-N-acetylglucosamine--N-acetylmuramyl-(pentapeptide) pyrophosphoryl-undecaprenol N-acetylglucosamine transferase**
155	SAL0000155	*argR_3*	94.7	Arginine repressor
**156**	**SAL0000156**	***group_5863***	**94.7**	**hypothetical protein**
157	SAL0000157	*flK*	94.7	Thioesterase superfamily protein
158	SAL0000158	*corA_1*	94.7	Magnesium transport protein CorA
159	SAL0000159	*group_5876*	94.7	Nicotinamide mononucleotide transporter
160	SAL0000160	*group_5880*	94.7	Putative GTP cyclohydrolase 1 type 2
161	SAL0000161	*arsC*	94.7	Arsenate reductase
162	SAL0000162	*group_5883*	94.7	hypothetical protein
163	SAL0000163	*artJ*	94.7	ABC transporter arginine-binding protein 1 precursor
**164**	**SAL0000164**	***group_5886***	**94.7**	**CsbD-like protein**
**165**	**SAL0000165**	***ybbH_2***	**94.7**	**putative HTH-type transcriptional regulator YbbH**
**166**	**SAL0000166**	***group_5902***	**94.7**	**acetyltransferase**
**167**	**SAL0000167**	***panE***	**94.7**	**2-dehydropantoate 2-reductase**
168	SAL0000168	*carA*	94.7	Carbamoyl-phosphate synthase small chain
169	SAL0000169	*group_5922*	94.7	hypothetical protein
**170**	**SAL0000170**	***group_5923***	**94.7**	**hypothetical protein**
**171**	**SAL0000171**	***atpB***	**94.7**	**ATP synthase subunit a**
172	SAL0000172	*group_5930*	94.7	putative acyltransferase
**173**	**SAL0000173**	***ybaK***	**94.7**	**Cys-tRNA(Pro)/Cys-tRNA(Cys) deacylase YbaK**
174	SAL0000174	*group_5940*	94.7	hypothetical protein
175	SAL0000175	*dgkA*	94.7	Undecaprenol kinase
176	SAL0000176	*ideR*	94.7	Iron-dependent repressor IdeR
177	SAL0000177	*group_5960*	94.7	coproporphyrinogen III oxidase
178	SAL0000178	*artP_2*	94.7	Arginine-binding extracellular protein ArtP precursor
179	SAL0000179	*dtd*	94.7	D-tyrosyl-tRNA(Tyr) deacylase
180	SAL0000180	*rsmE*	94.7	Ribosomal RNA small subunit methyltransferase E
181	SAL0000181	*fabZ*	94.7	3-hydroxyacyl-[acyl-carrier-protein] dehydratase FabZ

182	SAL0000182	*ecfT_1*	94.7	Energy-coupling factor transporter transmembrane protein EcfT
183	SAL0000183	*lrp*	94.7	Leucine-rich protein
**184**	**SAL0000184**	***group_6177***	**94.7**	**hypothetical protein**
185	SAL0000185	*rpoA*	94.7	DNA-directed RNA polymerase subunit alpha
**186**	**SAL0000186**	***rpsH***	**94.7**	**30S ribosomal protein S8**
187	SAL0000187	*proB*	94.7	Glutamate 5-kinase 1
188	SAL0000188	*copY*	94.7	Transcriptional repressor CopY
189	SAL0000189	*tsaE*	94.7	tRNA threonylcarbamoyladenosine biosynthesis protein TsaE
190	SAL0000190	*accA*	94.7	Acetyl-coenzyme A carboxylase carboxyl transferase subunit alpha
191	SAL0000191	*moeZ_1*	94.7	putative adenylyltransferase/sulfurtransferase MoeZ
192	SAL0000192	*csoR*	94.7	Copper-sensing transcriptional repressor CsoR
**193**	**SAL0000193**	***efp***	**94.7**	**Elongation factor P**
194	SAL0000194	*ssb_1*	94.7	Single-stranded DNA-binding protein ssb
195	SAL0000195	*rnhC*	94.7	Ribonuclease HIII
196	SAL0000196	*group_6229*	94.7	Helix-turn-helix
197	SAL0000197	*glnR*	94.7	HTH-type transcriptional regulator GlnR
198	SAL0000198	*ulaF*	94.7	L-ribulose-5-phosphate 4-epimerase UlaF
**199**	**SAL0000199**	***rplI***	**94.7**	**50S ribosomal protein L9**
200	SAL0000200	*albF*	94.7	Putative zinc protease AlbF
201	SAL0000201	*group_6265*	94.7	hypothetical protein
202	SAL0000202	*proX*	94.7	Prolyl-tRNA editing protein ProX
203	SAL0000203	*group_6271*	94.7	metal-dependent hydrolase
204	SAL0000204	*group_6280*	94.7	hypothetical protein
205	SAL0000205	*group_6284*	94.7	hypothetical protein
206	SAL0000206	*lipC*	94.7	Spore germination lipase LipC
207	SAL0000207	*recR*	94.7	Recombination protein RecR
208	SAL0000208	*yidA_4*	94.7	Sugar phosphatase YidA
209	SAL0000209	*paaI*	94.7	Acyl-coenzyme A thioesterase PaaI
210	SAL0000210	*dnaD*	94.7	DNA replication protein DnaD
211	SAL0000211	*rfbC*	94.7	putative dTDP-4-dehydrorhamnose 3,5-epimerase
212	SAL0000212	*cpsB*	94.7	Tyrosine-protein phosphatase CpsB
213	SAL0000213	*group_6317*	94.7	SNARE associated Golgi protein
214	SAL0000214	*ytrA*	94.7	HTH-type transcriptional repressor YtrA
**215**	**SAL0000215**	***group_6322***	**94.7**	**hypothetical protein**
216	SAL0000216	*group_6325*	94.7	hypothetical protein
217	SAL0000217	*group_6327*	94.7	hypothetical protein
218	SAL0000218	*group_6334*	94.7	Cupin domain protein
219	SAL0000219	*group_6335*	94.7	putative HTH-type transcriptional regulator
220	SAL0000220	*group_6336*	94.7	putative DNA-binding protein
221	SAL0000221	*xpt*	94.7	Xanthine phosphoribosyltransferase
222	SAL0000222	*group_6345*	94.7	GDSL-like Lipase/Acylhydrolase
223	SAL0000223	*bglA_2*	94.7	6-phospho-beta-glucosidase BglA
224	SAL0000224	*group_6351*	94.7	DegV domain-containing protein
225	SAL0000225	*group_6352*	94.7	hypothetical protein
226	SAL0000226	*cmk*	94.7	Cytidylate kinase
**227**	**SAL0000227**	***rpsU***	**94.7**	**30S ribosomal protein S21**
228	SAL0000228	*serB*	94.7	Phosphoserine phosphatase
229	SAL0000229	*gph_2*	94.7	Phosphorylated carbohydrates phosphatase
**230**	**SAL0000230**	***group_6366***	**94.7**	**hypothetical protein**
231	SAL0000231	*group_6367*	94.7	hypothetical protein
**232**	**SAL0000232**	***pspA***	**94.7**	**Phosphoserine phosphatase 1**
**233**	**SAL0000233**	***group_6369***	**94.7**	**hypothetical protein**
234	SAL0000234	*yhbU_2*	94.7	putative protease YhbU precursor
235	SAL0000235	*group_6372*	94.7	hypothetical protein
236	SAL0000236	*group_6373*	94.7	hypothetical protein
237	SAL0000237	*group_6374*	94.7	hypothetical protein
238	SAL0000238	*group_6375*	94.7	putative permease
**239**	**SAL0000239**	***group_6382***	**94.7**	**Uracil DNA glycosylase superfamily protein**
240	SAL0000240	*mgsR*	94.7	Regulatory protein MgsR
**241**	**SAL0000241**	***group_6386***	**94.7**	**CutC-like protein**
242	SAL0000242	*ribU*	94.7	Riboflavin transporter RibU
243	SAL0000243	*scpB*	94.7	Segregation and condensation protein B

244	SAL0000244	*lysA*	94.7	Diaminopimelate decarboxylase
**245**	**SAL0000245**	***nrdR***	**94.7**	**Transcriptional repressor NrdR**
246	SAL0000246	*group_6403*	94.7	hypothetical protein
247	SAL0000247	*ppaX*	94.7	Pyrophosphatase PpaX
248	SAL0000248	*rluD_1*	94.7	Ribosomal large subunit pseudouridine synthase D
249	SAL0000249	*group_6596*	94.7	hypothetical protein
250	SAL0000250	*ecfA2*	94.7	Energy-coupling factor transporter ATP-binding protein EcfA2
251	SAL0000251	*mecA*	94.7	Adapter protein MecA
252	SAL0000252	*hpdA*	94.7	4-hydroxyphenylacetate decarboxylase activating enzyme
253	SAL0000253	*znuC_1*	94.7	High-affinity zinc uptake system ATP-binding protein ZnuC
254	SAL0000254	*cydB*	94.7	Cytochrome bd-I ubiquinol oxidase subunit 2
255	SAL0000255	*rpmD*	94.7	50S ribosomal protein L30
**256**	**SAL0000256**	***rplX***	**94.7**	**50S ribosomal protein L24**
**257**	**SAL0000257**	***rpsQ***	**94.7**	**30S ribosomal protein S17**
258	SAL0000258	*rplP*	94.7	50S ribosomal protein L16
259	SAL0000259	*acpP_2*	94.7	Acyl carrier protein
**260**	**SAL0000260**	***group_6750***	**94.7**	**Putative protein phosphatase 2C-type**
**261**	**SAL0000261**	***ftsL***	**94.7**	**Cell division protein FtsL**
262	SAL0000262	*group_6752*	94.7	Enterocin A Immunity
**263**	**SAL0000263**	***yidA_3***	**94.7**	**Sugar phosphatase YidA**
264	SAL0000264	*rimP*	94.7	Ribosome maturation factor RimP
265	SAL0000265	*group_6756*	94.7	Phosphotransferase enzyme family protein
266	SAL0000266	*group_6757*	94.7	HIT-like protein
**267**	**SAL0000267**	***mprA***	**94.7**	**Transcriptional repressor MprA**
**268**	**SAL0000268**	***group_6761***	**94.7**	**hypothetical protein**
**269**	**SAL0000269**	***acpS***	**94.7**	**Holo-[acyl-carrier-protein] synthase**
**270**	**SAL0000270**	***rpsF***	**94.7**	**30S ribosomal protein S6**
**271**	**SAL0000271**	***group_6768***	**94.7**	**Colicin V production protein**
**272**	**SAL0000272**	***group_6769***	**94.7**	**hypothetical protein**
**273**	**SAL0000273**	***group_6770***	**94.7**	**hypothetical protein**
274	SAL0000274	*oppF*	94.7	Oligopeptide transport ATP-binding protein OppF
275	SAL0000275	*tabA*	94.7	Toxin-antitoxin biofilm protein TabA
276	SAL0000276	*group_6773*	94.7	DNA-binding transcriptional activator GutM
277	SAL0000277	*group_6774*	94.7	Endoribonuclease L-PSP
**278**	**SAL0000278**	***groS***	**94.7**	**10 kDa chaperonin**
279	SAL0000279	*cspC_2*	94.7	Cold shock protein CspC
280	SAL0000280	*arsR_2*	94.7	Arsenical resistance operon repressor
281	SAL0000281	*group_6801*	94.7	hypothetical protein
**282**	**SAL0000282**	***rlmH***	**94.7**	**Ribosomal RNA large subunit methyltransferase H**
**283**	**SAL0000283**	***group_6803***	**94.7**	**Septum formation initiator**
284	SAL0000284	*group_6804*	94.7	GDSL-like Lipase/Acylhydrolase
285	SAL0000285	*group_6814*	94.7	hypothetical protein
286	SAL0000286	*group_6815*	94.7	hypothetical protein
287	SAL0000287	*group_6816*	94.7	hypothetical protein
**288**	**SAL0000288**	***group_6817***	**94.7**	**hypothetical protein**
289	SAL0000289	*group_6819*	94.7	hypothetical protein
290	SAL0000290	*group_6822*	94.7	hypothetical protein
**291**	**SAL0000291**	***nudG***	**94.7**	**CTP pyrophosphohydrolase**
292	SAL0000292	*group_6826*	94.7	D-Ala-teichoic acid biosynthesis protein
293	SAL0000293	*group_6828*	94.7	hypothetical protein
294	SAL0000294	*group_6829*	94.7	Acyl-ACP thioesterase
295	SAL0000295	*group_6834*	94.7	hypothetical protein
296	SAL0000296	*plsY*	94.7	Glycerol-3-phosphate acyltransferase
297	SAL0000297	*group_6836*	94.7	hypothetical protein
298	SAL0000298	*panT*	94.7	Pantothenic acid transporter PanT
**299**	**SAL0000299**	***nrdD_1***	**94.7**	**Anaerobic ribonucleoside-triphosphate reductase**
300	SAL0000300	*maa*	94.7	Maltose O-acetyltransferase
**301**	**SAL0000301**	***group_6841***	**94.7**	**hypothetical protein**
302	SAL0000302	*group_6842*	94.7	hypothetical protein
303	SAL0000303	*azr_4*	94.7	NADPH azoreductase
304	SAL0000304	*group_6846*	94.7	hypothetical protein
305	SAL0000305	*yhhX*	94.7	putative oxidoreductase YhhX
306	SAL0000306	*folB*	94.7	Dihydroneopterin aldolase
**307**	**SAL0000307**	***immR_1***	**94.7**	**HTH-type transcriptional regulator ImmR**

308	SAL0000308	*group_6855*	94.7	Aldose 1-epimerase
309	SAL0000309	*group_6856*	94.7	hypothetical protein
310	SAL0000310	*licT_3*	94.7	Transcription antiterminator LicT
311	SAL0000311	*group_6859*	94.7	Muramidase-2 precursor
312	SAL0000312	*rimM*	94.7	Ribosome maturation factor RimM
**313**	**SAL0000313**	***rpsP***	**94.7**	**30S ribosomal protein S16**
314	SAL0000314	*pyrB*	94.7	Aspartate carbamoyltransferase
315	SAL0000315	*rpmI*	94.7	50S ribosomal protein L35
**316**	**SAL0000316**	***group_6865***	**94.7**	**hypothetical protein**
317	SAL0000317	*atpF*	94.7	ATP synthase subunit b
**318**	**SAL0000318**	***rplS***	**94.7**	**50S ribosomal protein L19**
319	SAL0000319	*group_6870*	94.7	hypothetical protein
320	SAL0000320	*group_6871*	94.7	putative metallo-hydrolase
**321**	**SAL0000321**	***glpQ1***	**94.7**	**putative glycerophosphoryl diester phosphodiesterase 1**
322	SAL0000322	*group_6873*	94.7	Glycosyl hydrolases family 25
323	SAL0000323	*thiT*	94.7	Thiamine transporter ThiT
324	SAL0000324	*group_6875*	94.7	hypothetical protein
325	SAL0000325	*phoP*	94.7	Alkaline phosphatase synthesis transcriptional regulatory protein PhoP
**326**	**SAL0000326**	***group_6878***	**94.7**	**Glyoxalase-like domain protein**
327	SAL0000327	*yycJ*	94.7	Putative metallo-hydrolase YycJ
328	SAL0000328	*group_6881*	94.7	cellobiose phosphotransferase system IIB component
329	SAL0000329	*gloA*	94.7	Lactoylglutathione lyase
330	SAL0000330	*group_6888*	94.7	inosine 5'-monophosphate dehydrogenase
331	SAL0000331	*group_6889*	94.7	hypothetical protein
332	SAL0000332	*clpP*	94.7	ATP-dependent Clp protease proteolytic subunit
333	SAL0000333	*group_6892*	94.7	hypothetical protein
334	SAL0000334	*group_6894*	94.7	Putative membrane protein insertion efficiency factor
335	SAL0000335	*acyP*	94.7	Acylphosphatase
336	SAL0000336	*group_6896*	94.7	hypothetical protein
**337**	**SAL0000337**	***rsfS***	**94.7**	**Ribosomal silencing factor RsfS**
338	SAL0000338	*group_6898*	94.7	GTPase YlqF
339	SAL0000339	*group_6927*	94.7	hypothetical protein
340	SAL0000340	*yugI*	94.7	General stress protein 13
341	SAL0000341	*group_7158*	94.7	YtxH-like protein
342	SAL0000342	*yjiR*	94.7	putative HTH-type transcriptional regulator YjiR
**343**	**SAL0000343**	***group_7214***	**94.7**	**hypothetical protein**
344	SAL0000344	*rplC*	94.7	50S ribosomal protein L3
**345**	**SAL0000345**	***group_7350***	**94.7**	**hypothetical protein**
**346**	**SAL0000346**	***rpmB***	**94.7**	**50S ribosomal protein L28**
347	SAL0000347	*infA*	94.7	Translation initiation factor IF-1
348	SAL0000348	*rplR*	94.7	50S ribosomal protein L18
349	SAL0000349	*rpsZ*	94.7	30S ribosomal protein S14 type Z
350	SAL0000350	*recO*	94.7	DNA repair protein RecO
351	SAL0000351	*rpoZ*	94.7	DNA-directed RNA polymerase subunit omega
352	SAL0000352	*gmk*	94.7	Guanylate kinase
353	SAL0000353	*gpsB*	94.7	Cell cycle protein GpsB
**354**	**SAL0000354**	***group_7391***	**94.7**	**hypothetical protein**
355	SAL0000355	*group_7393*	94.7	hypothetical protein
356	SAL0000356	*trmB*	94.7	tRNA (guanine-N(7)-)-methyltransferase
357	SAL0000357	*yidA_2*	94.7	Sugar phosphatase YidA
358	SAL0000358	*group_7396*	94.7	hypothetical protein
359	SAL0000359	*accB*	94.7	Biotin carboxyl carrier protein of acetyl-CoA carboxylase
**360**	**SAL0000360**	***acpP_1***	**94.7**	**Acyl carrier protein**
361	SAL0000361	*group_7400*	94.7	Cold shock protein CspC
362	SAL0000362	*gatC_1*	94.7	Glutamyl-tRNA(Gln) amidotransferase subunit C
**363**	**SAL0000363**	***group_7406***	**94.7**	**hypothetical protein**
**364**	**SAL0000364**	***sipU***	**94.7**	**Signal peptidase I U**
365	SAL0000365	*group_7409*	94.7	hypothetical protein
366	SAL0000366	*group_7420*	94.7	hypothetical protein
367	SAL0000367	*ulaB*	94.7	Ascorbate-specific phosphotransferase enzyme IIB component
368	SAL0000368	*group_7426*	94.7	hypothetical protein
369	SAL0000369	*group_7427*	94.7	preprotein translocase subunit SecE
**370**	**SAL0000370**	***ctsR***	**94.7**	**Transcriptional regulator CtsR**

**371**	**SAL0000371**	***rpmF***	**94.7**	**50S ribosomal protein L32**
372	SAL0000372	*rpmGA*	94.7	50S ribosomal protein L33 1
373	SAL0000373	*fabR*	94.7	HTH-type transcriptional repressor FabR
374	SAL0000374	*ecfA1*	94.7	Energy-coupling factor transporter ATP-binding protein EcfA1
375	SAL0000375	*group_7485*	94.7	hypothetical protein
**376**	**SAL0000376**	***hslR***	**94.7**	**Heat shock protein 15**
**377**	**SAL0000377**	***hpt***	**94.7**	**Hypoxanthine-guanine phosphoribosyltransferase**
378	SAL0000378	*rbsD*	94.7	D-ribose pyranase
379	SAL0000379	*group_7502*	94.7	YGGT family protein
380	SAL0000380	*nrdF1*	94.7	Ribonucleoside-diphosphate reductase subunit beta nrdF1
381	SAL0000381	*nrdH*	94.7	Glutaredoxin-like protein NrdH
382	SAL0000382	*group_7511*	94.7	Nucleoside 2-deoxyribosyltransferase
383	SAL0000383	*pdg*	94.7	Ultraviolet N-glycosylase/AP lyase
**384**	**SAL0000384**	***group_7513***	**94.7**	**hypothetical protein**
**385**	**SAL0000385**	***mutX***	**94.7**	**8-oxo-dGTP diphosphatase**
386	SAL0000386	*group_7517*	94.7	Putative NAD(P)H nitroreductase
**387**	**SAL0000387**	***spsB***	**94.7**	**Signal peptidase IB**
388	SAL0000388	*group_7528*	94.7	hypothetical protein
389	SAL0000389	*group_7529*	94.7	DNA-binding transcriptional repressor MngR
390	SAL0000390	*tpx*	94.7	putative thiol peroxidase
391	SAL0000391	*pstB3_2*	94.7	Phosphate import ATP-binding protein PstB 3
392	SAL0000392	*arlS_2*	94.7	Signal transduction histidine-protein kinase ArlS
**393**	**SAL0000393**	***thyA***	**94.7**	**Thymidylate synthase**
**394**	**SAL0000394**	***lacR_1***	**94.7**	**Lactose phosphotransferase system repressor**
**395**	**SAL0000395**	***group_7546***	**94.7**	**hypothetical protein**
**396**	**SAL0000396**	***rpmA***	**94.7**	**50S ribosomal protein L27**
397	SAL0000397	*group_7548*	94.7	hypothetical protein
398	SAL0000398	*group_7552*	94.7	OsmC-like protein
**399**	**SAL0000399**	***group_7553***	**94.7**	**DNA-directed RNA polymerase subunit beta**
400	SAL0000400	*atpC*	94.7	ATP synthase epsilon chain
**401**	**SAL0000401**	***cspC_3***	**94.7**	**Cold shock protein CspC**
**402**	**SAL0000402**	***yccF***	**94.7**	**Inner membrane protein YccF**
403	SAL0000403	*group_7560*	94.7	hypothetical protein
**404**	**SAL0000404**	***mgs***	**94.7**	**Alpha-monoglucosyldiacylglycerol synthase**
**405**	**SAL0000405**	***group_7566***	**94.7**	**preprotein translocase subunit SecG**
406	SAL0000406	*group_7572*	94.7	hypothetical protein
407	SAL0000407	*group_7573*	94.7	hypothetical protein
**408**	**SAL0000408**	***group_7574***	**94.7**	**hypothetical protein**
409	SAL0000409	*sdpR*	94.7	Transcriptional repressor SdpR
410	SAL0000410	*rsmI*	94.7	Ribosomal RNA small subunit methyltransferase I
**411**	**SAL0000411**	***group_7577***	**94.7**	**Initiation-control protein YabA**
412	SAL0000412	*ykuL*	94.7	CBS domain-containing protein YkuL
**413**	**SAL0000413**	***nadD***	**94.7**	**Nicotinate-nucleotide adenylyltransferase**
414	SAL0000414	*group_7583*	94.7	RNA-binding protein
415	SAL0000415	*group_7584*	94.7	hypothetical protein
416	SAL0000416	*rluB*	94.7	Ribosomal large subunit pseudouridine synthase B

Supplementary File S2.D. Venn diagram output with ellipses from wgMLST (whole-genome multilocus sequence typing). It illustrates the allelic overlap among the compared bacterial genomes, marking the inclusion of loci when 100, 90, 70, and 50% of the isolates are included in the analysis. Each ellipse represents a group of isolates (Occ100, green; Occ90, blue; Occ70, purple; Occ50, yellow), and the overlapping regions indicate the number of shared loci (genes) between isolates.

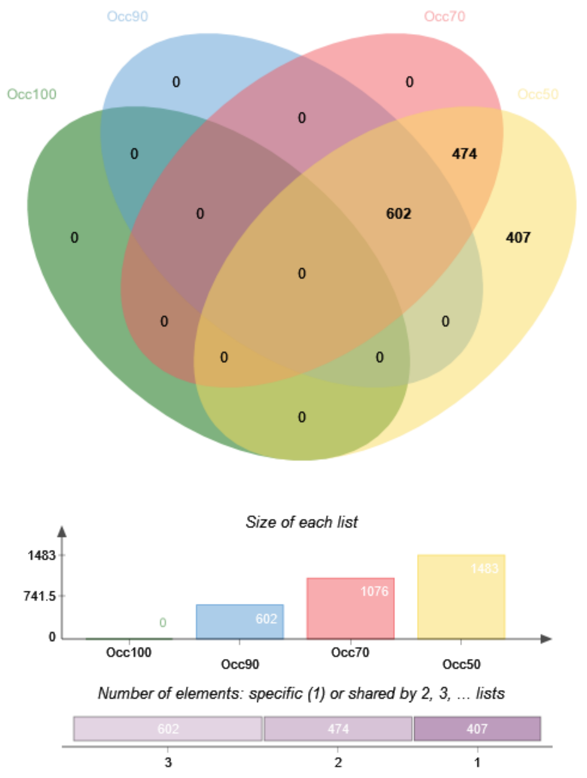

Supplement: Supplementary file 1 [file antibiotics-14-01059-s001.zip › antibiotics-3895307-supplementary/Supplementary File S2.pdf]
