# Supplementary material for: Genomic Characterization and Antimicrobial Resistance Profile of Streptococcus uberis Strains Isolated from Cows with Mastitis from Northwestern Spain
Source: Antibiotics (Basel). 2025 Oct 23;14(11):1059. doi: 10.3390/antibiotics14111059 (PMC12649216; doi:10.3390/antibiotics14111059)

Supplementary File S3.A. Venn diagram output with ellipses from wgMLST. It illustrates the composition of the whole genome, marking the inclusion of loci when 100, 90, 70, and 50% of the isolates are included (Occ100, green; Occ90, blue; Occ70, purple; Occ50, yellow). Thirty-six isolates were studied, i.e., without reference strains (*S. uberis* and *Escherichia coli*).

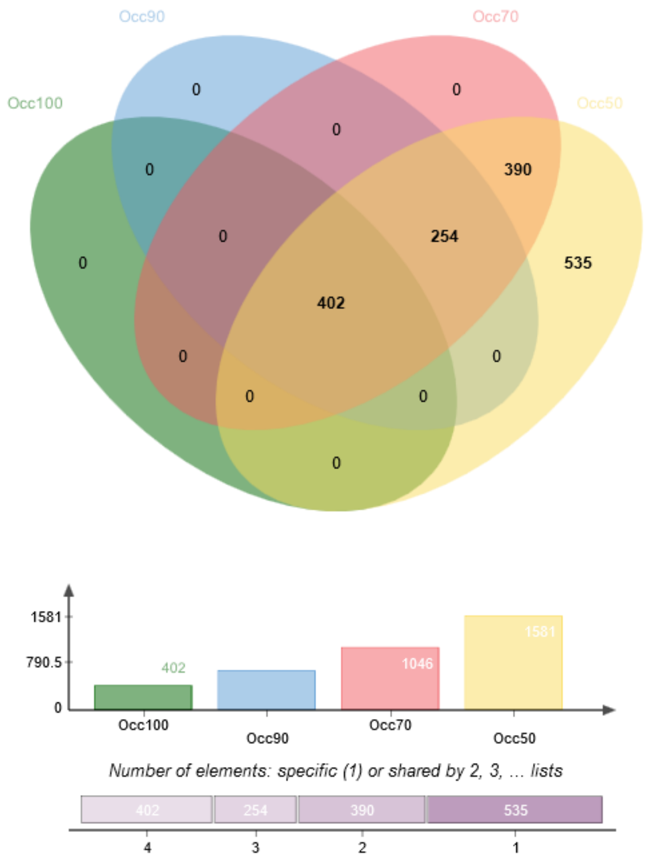

Supplementary File S3.B. Genetic relatedness tree from wgMLST. No reference strains included.

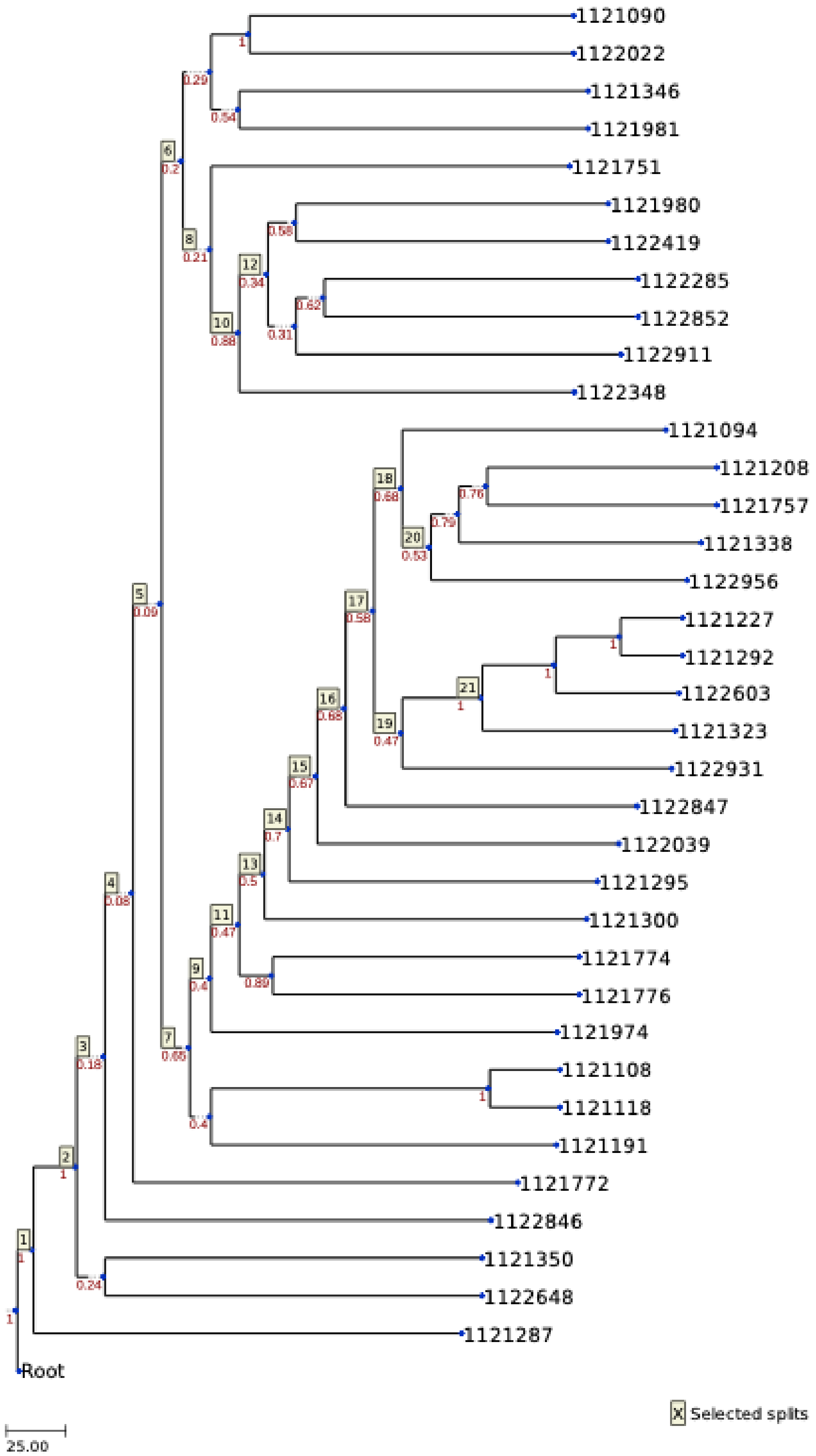

Supplementary File S3.C. The most discriminatory loci (402 with >95% of strains occurrence) from wgMLST. The 88 highly discriminatory loci are also shown (in light green). Thirty-six isolates were studied (no reference strains included).

#	Locus	Splits	Gene name	Occurrence (%)	Annotation
1	SAL0000001	-	group_10206	100.0	hypothetical protein
2	SAL0000002	-	group_10209	100.0	Initiation-control protein YabA
3	SAL0000003	7,10	group_10210	100.0	hypothetical protein
4	SAL0000004	-	gatC_1	100.0	Glutamyl-tRNA(Gln) amidotransferase subunit C
5	SAL0000005	-	rpmB	100.0	50S ribosomal protein L28
6	SAL0000006	-	rpmGA	100.0	50S ribosomal protein L33 1
7	SAL0000007	-	acpP_1	100.0	Acyl carrier protein
8	SAL0000008	1	hslR	100.0	Heat shock protein 15
9	SAL0000009	-	nrdH	100.0	Glutaredoxin-like protein NrdH
10	SAL0000010	1,13	group_10217	100.0	hypothetical protein
11	SAL0000011	7,13	rpsZ	100.0	30S ribosomal protein S14 type Z
12	SAL0000012	-	group_10219	100.0	hypothetical protein
13	SAL0000013	2,12	infA	100.0	Translation initiation factor IF-1
14	SAL0000014	2	group_10221	100.0	RNA-binding protein
15	SAL0000015	-	nrdF1	100.0	Ribonucleoside-diphosphate reductase subunit beta nrdF1
16	SAL0000016	10	mgs	100.0	Alpha-monoglucosyldiacylglycerol synthase
17	SAL0000017	13	ctsR	100.0	Transcriptional regulator CtsR
18	SAL0000018	-	group_10225	100.0	OsmC-like protein
19	SAL0000019	-	fabR	100.0	HTH-type transcriptional repressor FabR
20	SAL0000020	-	group_10227	100.0	hypothetical protein
21	SAL0000021	18	group_10228	100.0	hypothetical protein
22	SAL0000022	-	group_10229	100.0	DNA-binding transcriptional repressor MngR
23	SAL0000023	20	spsB	100.0	Signal peptidase IB
24	SAL0000024	-	yidA_2	100.0	Sugar phosphatase YidA
25	SAL0000025	-	rpmH	100.0	50S ribosomal protein L34
26	SAL0000026	-	gmk	100.0	Guanylate kinase
27	SAL0000027	-	rplR	100.0	50S ribosomal protein L18
28	SAL0000028	11	pdg	100.0	Ultraviolet N-glycosylase/AP lyase
29	SAL0000029	-	group_10240	100.0	hypothetical protein
30	SAL0000030	1	rpoZ	100.0	DNA-directed RNA polymerase subunit omega
31	SAL0000031	-	gpsB	100.0	Cell cycle protein GpsB
32	SAL0000032	-	ykuL	100.0	CBS domain-containing protein YkuL
33	SAL0000033	-	trmB	100.0	tRNA (guanine-N(7)-)-methyltransferase
34	SAL0000034	12	hpt	100.0	Hypoxanthine-guanine phosphoribosyltransferase
35	SAL0000035	-	asnA	100.0	Aspartate--ammonia ligase
36	SAL0000036	-	group_10250	100.0	hypothetical protein
37	SAL0000037	-	mutX	100.0	8-oxo-dGTP diphosphatase
38	SAL0000038	-	group_10254	100.0	Nucleoside 2-deoxyribosyltransferase
39	SAL0000039	21	rsmI	100.0	Ribosomal RNA small subunit methyltransferase I
40	SAL0000040	-	rpmA	100.0	50S ribosomal protein L27
41	SAL0000041	-	cspC_2	100.0	Cold shock protein CspC
42	SAL0000042	4	group_10264	100.0	hypothetical protein
43	SAL0000043	-	rluB	100.0	Ribosomal large subunit pseudouridine synthase B
44	SAL0000044	-	ulaB	100.0	Ascorbate-specific phosphotransferase enzyme IIB component
45	SAL0000045	-	arlS_1	100.0	Signal transduction histidine-protein kinase ArlS
46	SAL0000046	-	group_10272	100.0	preprotein translocase subunit SecG
47	SAL0000047	-	gltC	100.0	HTH-type transcriptional regulator GltC
48	SAL0000048	-	group_10274	100.0	YGGT family protein
49	SAL0000049	10	group_10275	100.0	hypothetical protein
50	SAL0000050	20	tpx	100.0	putative thiol peroxidase
51	SAL0000051	-	group_10279	100.0	hypothetical protein
52	SAL0000052	17	atpC	100.0	ATP synthase epsilon chain
53	SAL0000053	-	group_10281	100.0	hypothetical protein
54	SAL0000054	-	cspC_3	100.0	Cold shock protein CspC
55	SAL0000055	14,21	thyA	100.0	Thymidylate synthase
56	SAL0000056	-	recO	100.0	DNA repair protein RecO
57	SAL0000057	-	ecfA1	100.0	Energy-coupling factor transporter ATP-binding protein EcfA1

58	SAL0000058	-	sdpR	100.0	Transcriptional repressor SdpR
59	SAL0000059	-	group_10289	100.0	hypothetical protein
60	SAL0000060	-	rbsD	100.0	D-ribose pyranase
61	SAL0000061	-	pstB3_2	100.0	Phosphate import ATP-binding protein PstB 3
62	SAL0000062	4	rpmF	100.0	50S ribosomal protein L32
63	SAL0000063	-	group_10295	100.0	preprotein translocase subunit SecE
64	SAL0000064	-	group_10296	100.0	hypothetical protein
65	SAL0000065	-	nadD	100.0	Nicotinate-nucleotide adenylyltransferase
66	SAL0000066	-	group_10299	100.0	Putative phosphatase
67	SAL0000067	-	lacR_1	100.0	Lactose phosphotransferase system repressor
68	SAL0000068	17	yccF	100.0	Inner membrane protein YccF
69	SAL0000069	-	group_10302	100.0	hypothetical protein
70	SAL0000070	-	group_10303	100.0	hypothetical protein
71	SAL0000071	17	group_10304	100.0	DNA-directed RNA polymerase subunit beta
72	SAL0000072	-	group_10305	100.0	Putative NAD(P)H nitroreductase
73	SAL0000073	-	group_10306	100.0	hypothetical protein
74	SAL0000074	20	accB	100.0	Biotin carboxyl carrier protein of acetyl-CoA carboxylase
75	SAL0000075	-	serC	100.0	Phosphoserine aminotransferase
76	SAL0000076	-	group_1610	100.0	hypothetical protein
77	SAL0000077	-	group_1681	100.0	Putative acetyltransferase
78	SAL0000078	-	clpX	100.0	hypothetical protein
79	SAL0000079	5	group_1981	100.0	hypothetical protein
80	SAL0000080	9	rplL	100.0	50S ribosomal protein L7/L12
81	SAL0000081	-	gluP	100.0	Rhomboid protease GluP
82	SAL0000082	-	rplO	100.0	50S ribosomal protein L15
83	SAL0000083	5	rpmC	100.0	50S ribosomal protein L29
84	SAL0000084	-	group_3392	100.0	hypothetical protein
85	SAL0000085	-	group_3406	100.0	pyridoxal phosphate (PLP) phosphatase
86	SAL0000086	-	obg	100.0	GTPase ObgE
87	SAL0000087	-	group_3757	100.0	hypothetical protein
88	SAL0000088	-	azr_2	100.0	NADPH azoreductase
89	SAL0000089	-	group_4082	100.0	hypothetical protein
90	SAL0000090	-	group_4114	100.0	hypothetical protein
91	SAL0000091	-	group_4196	100.0	hypothetical protein
92	SAL0000092	-	adhR	100.0	HTH-type transcriptional regulator AdhR
93	SAL0000093	-	group_4221	100.0	hypothetical protein
94	SAL0000094	-	group_4264	100.0	hypothetical protein
95	SAL0000095	19	mscL	100.0	Large-conductance mechanosensitive channel
96	SAL0000096	6	ftsX	100.0	Cell division protein FtsX
97	SAL0000097	-	hprK	100.0	HPr kinase/phosphorylase
98	SAL0000098	5	rpsT	100.0	30S ribosomal protein S20
99	SAL0000099	-	group_4420	100.0	hypothetical protein
100	SAL0000100	-	group_4433	100.0	hypothetical protein
101	SAL0000101	-	group_4457	100.0	Ribosome-associated factor Y
102	SAL0000102	-	group_4478	100.0	hypothetical protein
103	SAL0000103	20	group_4488	100.0	hypothetical protein
104	SAL0000104	-	group_4493	100.0	hypothetical protein
105	SAL0000105	-	moeZ_2	100.0	putative adenylyltransferase/sulfurtransferase MoeZ
106	SAL0000106	21	polC_1	100.0	DNA polymerase III PolC-type
107	SAL0000107	-	spxA_1	100.0	Regulatory protein Spx
108	SAL0000108	-	nudC	100.0	NADH pyrophosphatase
109	SAL0000109	5	pgsA	100.0	CDP-diacylglycerol--glycerol-3-phosphate 3-phosphatidyltransferase
110	SAL0000110	-	group_4623	100.0	hypothetical protein
111	SAL0000111	-	yvoA_2	100.0	HTH-type transcriptional repressor YvoA
112	SAL0000112	-	glgA	100.0	Glycogen synthase
113	SAL0000113	-	msrA	100.0	Peptide methionine sulfoxide reductase MsrA
114	SAL0000114	-	trxC	100.0	Thioredoxin-2
115	SAL0000115	-	dexB	100.0	Glucan 1,6-alpha-glucosidase
116	SAL0000116	-	group_4883	100.0	Single-stranded DNA-binding protein ssb
117	SAL0000117	14	adk	100.0	Adenylate kinase
118	SAL0000118	-	tadA_1	100.0	tRNA-specific adenosine deaminase
119	SAL0000119	16	group_4930	100.0	Nucleoid-associated protein
120	SAL0000120	-	murR_2	100.0	HTH-type transcriptional regulator MurR
121	SAL0000121	-	group_4953	100.0	hypothetical protein
122	SAL0000122	-	group_4954	100.0	hypothetical protein
123	SAL0000123	-	skc	100.0	Streptokinase C precursor

124	SAL0000124	-	pat	100.0	Phosphinothricin N-acetyltransferase
125	SAL0000125	-	lptB_1	100.0	Lipopolysaccharide export system ATP-binding protein LptB
126	SAL0000126	-	argR_3	100.0	Arginine repressor
127	SAL0000127	15	yniC	100.0	Phosphorylated carbohydrates phosphatase
128	SAL0000128	-	group_5039	100.0	hypothetical protein
129	SAL0000129	-	mtrR	100.0	HTH-type transcriptional regulator MtrR
130	SAL0000130	-	group_5089	100.0	FtsX-like permease family protein
131	SAL0000131	-	gph	100.0	Phosphoglycolate phosphatase
132	SAL0000132	-	czcO	100.0	putative oxidoreductase CzcO
133	SAL0000133	-	feuB	100.0	Iron-uptake system permease protein FeuB
134	SAL0000134	-	group_5127	100.0	hypothetical protein
135	SAL0000135	-	trmL	100.0	Putative tRNA (cytidine(34)-2'-O)-methyltransferase
136	SAL0000136	-	group_5282	100.0	HTH-type transcriptional regulator MurR
137	SAL0000137	-	ecfT_1	100.0	Energy-coupling factor transporter transmembrane protein EcfT
138	SAL0000138	-	group_5328	100.0	preprotein translocase subunit YajC
139	SAL0000139	-	prmA	100.0	Ribosomal protein L11 methyltransferase
140	SAL0000140	-	group_5344	100.0	preprotein translocase subunit SecY
141	SAL0000141	-	purC	100.0	Phosphoribosylaminoimidazole-succinocarboxamide synthase
142	SAL0000142	-	mreC	100.0	Cell shape-determining protein MreC precursor
143	SAL0000143	-	luxS	100.0	S-ribosylhomocysteine lyase
144	SAL0000144	-	recU	100.0	Holliday junction resolvase RecU
145	SAL0000145	15	glyQ	100.0	Glycine--tRNA ligase alpha subunit
146	SAL0000146	-	group_5374	100.0	copper exporting ATPase
147	SAL0000147	-	gmuF	100.0	putative mannose-6-phosphate isomerase GmuF
148	SAL0000148	-	degA_2	100.0	HTH-type transcriptional regulator DegA
149	SAL0000149	13	group_5387	100.0	hypothetical protein
150	SAL0000150	-	thiN	100.0	Thiamine pyrophosphokinase
151	SAL0000151	-	dut	100.0	Deoxyuridine 5'-triphosphate nucleotidohydrolase
152	SAL0000152	-	azr_1	100.0	NADPH azoreductase
153	SAL0000153	-	group_5419	100.0	hypothetical protein
154	SAL0000154	17	yrrK	100.0	Putative Holliday junction resolvase
155	SAL0000155	-	smc_2	100.0	Chromosome partition protein Smc
156	SAL0000156	-	group_5436	100.0	hypothetical protein
157	SAL0000157	-	spo0C	100.0	Chromosome-partitioning protein Spo0J
158	SAL0000158	-	tlyA	100.0	Hemolysin A
159	SAL0000159	-	naiP	100.0	Putative niacin/nicotinamide transporter NaiP
160	SAL0000160	-	group_5472	100.0	Nicotinamide mononucleotide transporter
161	SAL0000161	-	group_5480	100.0	Cupin domain protein
162	SAL0000162	-	group_5483	100.0	hypothetical protein
163	SAL0000163	-	group_5488	100.0	hypothetical protein
164	SAL0000164	-	coaBC_1	100.0	Coenzyme A biosynthesis bifunctional protein CoaBC
165	SAL0000165	-	cynR_2	100.0	HTH-type transcriptional regulator CynR
166	SAL0000166	-	group_5518	100.0	hypothetical protein
167	SAL0000167	-	vioD	100.0	Capreomycidine synthase
168	SAL0000168	-	endA	100.0	DNA-entry nuclease
169	SAL0000169	-	group_5559	100.0	hypothetical protein
170	SAL0000170	-	mutM	100.0	Formamidopyrimidine-DNA glycosylase
171	SAL0000171	15	sdpI	100.0	Immunity protein SdpI
172	SAL0000172	-	group_5597	100.0	hypothetical protein
173	SAL0000173	-	tmk	100.0	Thymidylate kinase
174	SAL0000174	-	ytgP_1	100.0	putative cell division protein YtgP
175	SAL0000175	-	group_5609	100.0	putative acetyltransferase
176	SAL0000176	-	group_5612	100.0	Phosphorylated carbohydrates phosphatase
177	SAL0000177	-	group_5621	100.0	Deoxyguanosine kinase
178	SAL0000178	-	metQ	100.0	D-methionine-binding lipoprotein MetQ precursor
179	SAL0000179	-	yumC	100.0	Ferredoxin--NADP reductase 2
180	SAL0000180	-	group_5638	100.0	Helix-turn-helix
181	SAL0000181	-	rlmCD_1	100.0	23S rRNA (uracil-C(5))-methyltransferase RlmCD
182	SAL0000182	-	group_5709	100.0	Flavodoxin
183	SAL0000183	-	ywpJ	100.0	Putative phosphatase YwpJ
184	SAL0000184	-	mhqR	100.0	HTH-type transcriptional regulator MhqR
185	SAL0000185	-	group_5748	100.0	hypothetical protein
186	SAL0000186	16	group_5749	100.0	hypothetical protein
187	SAL0000187	-	group_5755	100.0	hypothetical protein
188	SAL0000188	-	murR_1	100.0	HTH-type transcriptional regulator MurR

189	SAL0000189	-	group_5772	100.0	hypothetical protein
190	SAL0000190	-	rplF	100.0	50S ribosomal protein L6
191	SAL0000191	-	rplN	100.0	50S ribosomal protein L14
192	SAL0000192	-	rpsC	100.0	30S ribosomal protein S3
193	SAL0000193	-	rplW	100.0	50S ribosomal protein L23
194	SAL0000194	-	group_5785	100.0	DNA utilization protein GntX
195	SAL0000195	-	rsmB	100.0	Ribosomal RNA small subunit methyltransferase B
196	SAL0000196	-	group_5790	100.0	hypothetical protein
197	SAL0000197	-	group_5804	100.0	Bacterial ABC transporter protein EcsB
198	SAL0000198	18	group_5808	100.0	Acyltransferase family protein
199	SAL0000199	8	lytG	100.0	Exo-glucosaminidase LytG precursor
200	SAL0000200	-	ccpN	100.0	Transcriptional repressor CcpN
201	SAL0000201	-	group_5822	100.0	hypothetical protein
202	SAL0000202	-	bglA_1	100.0	Aryl-phospho-beta-D-glucosidase BglA
203	SAL0000203	-	rpsN2	100.0	Alternate 30S ribosomal protein S14
204	SAL0000204	-	tsaB	100.0	tRNA threonylcarbamoyladenosine biosynthesis protein TsaB
205	SAL0000205	-	purR	100.0	Pur operon repressor
206	SAL0000206	-	gtaB	100.0	UTP--glucose-1-phosphate uridylyltransferase
207	SAL0000207	-	group_5833	100.0	PTS system glucitol/sorbitol-specific transporter subunit IIA
208	SAL0000208	-	licR_2	100.0	putative licABCH operon regulator
209	SAL0000209	-	ulaF	100.0	L-ribulose-5-phosphate 4-epimerase UlaF
210	SAL0000210	-	ulaC_1	100.0	Ascorbate-specific phosphotransferase enzyme IIA component
211	SAL0000211	7,8	rluC	100.0	Ribosomal large subunit pseudouridine synthase C
212	SAL0000212	-	sdhB	100.0	L-serine dehydratase, beta chain
213	SAL0000213	-	glcU_2	100.0	putative glucose uptake protein GlcU
214	SAL0000214	-	tilS	100.0	tRNA(Ile)-lysidine synthase
215	SAL0000215	16	group_5878	100.0	molybdopterin biosynthesis protein MoeB
216	SAL0000216	-	murG	100.0	UDP-N-acetylglucosamine--N-acetylmuramyl-(pentapeptide) pyrophosphoryl-undecaprenol N-acetylglucosamine transferase
217	SAL0000217	-	group_5881	100.0	hypothetical protein
218	SAL0000218	-	flK	100.0	Thioesterase superfamily protein
219	SAL0000219	-	corA_2	100.0	Magnesium transport protein CorA
220	SAL0000220	-	group_5899	100.0	Putative GTP cyclohydrolase 1 type 2
221	SAL0000221	-	arsC	100.0	Arsenate reductase
222	SAL0000222	-	group_5904	100.0	hypothetical protein
223	SAL0000223	-	artJ	100.0	ABC transporter arginine-binding protein 1 precursor
224	SAL0000224	-	group_5907	100.0	CsbD-like protein
225	SAL0000225	-	group_5908	100.0	hypothetical protein
226	SAL0000226	-	ybbH_2	100.0	putative HTH-type transcriptional regulator YbbH
227	SAL0000227	3,9	group_5924	100.0	acetyltransferase
228	SAL0000228	-	carA	100.0	Carbamoyl-phosphate synthase small chain
229	SAL0000229	-	group_5947	100.0	hypothetical protein
230	SAL0000230	-	group_5948	100.0	hypothetical protein
231	SAL0000231	16	atpB	100.0	ATP synthase subunit a
232	SAL0000232	-	group_5952	100.0	putative acyltransferase
233	SAL0000233	-	ybaK	100.0	Cys-tRNA(Pro)/Cys-tRNA(Cys) deacylase YbaK
234	SAL0000234	-	group_5960	100.0	hypothetical protein
235	SAL0000235	-	group_5961	100.0	hypothetical protein
236	SAL0000236	-	group_5965	100.0	putative permease
237	SAL0000237	21	dgkA	100.0	Undecaprenol kinase
238	SAL0000238	11	ideR	100.0	Iron-dependent repressor IdeR
239	SAL0000239	-	group_5986	100.0	coproporphyrinogen III oxidase
240	SAL0000240	-	artP_2	100.0	Arginine-binding extracellular protein ArtP precursor
241	SAL0000241	-	dtd	100.0	D-tyrosyl-tRNA(Tyr) deacylase
242	SAL0000242	-	rsmE	100.0	Ribosomal RNA small subunit methyltransferase E
243	SAL0000243	-	fabZ	100.0	3-hydroxyacyl-[acyl-carrier-protein] dehydratase FabZ
244	SAL0000244	-	lrp	100.0	Leucine-rich protein
245	SAL0000245	-	group_6195	100.0	hypothetical protein
246	SAL0000246	-	manY_1	100.0	Mannose permease IIC component
247	SAL0000247	-	rpoA	100.0	DNA-directed RNA polymerase subunit alpha
248	SAL0000248	8	rpsH	100.0	30S ribosomal protein S8
249	SAL0000249	-	proB	100.0	Glutamate 5-kinase 1
250	SAL0000250	-	copY	100.0	Transcriptional repressor CopY
251	SAL0000251	-	tsaE	100.0	tRNA threonylcarbamoyladenosine biosynthesis protein TsaE

252	SAL0000252	-	accA	100.0	Acetyl-coenzyme A carboxylase carboxyl transferase subunit alpha
253	SAL0000253	-	moeZ_1	100.0	putative adenylyltransferase/sulfurtransferase MoeZ
254	SAL0000254	10	csoR	100.0	Copper-sensing transcriptional repressor CsoR
255	SAL0000255	-	efp	100.0	Elongation factor P
256	SAL0000256	-	ssb_1	100.0	Single-stranded DNA-binding protein ssb
257	SAL0000257	-	rnhC	100.0	Ribonuclease HIII
258	SAL0000258	-	group_6243	100.0	Helix-turn-helix
259	SAL0000259	10	glnR	100.0	HTH-type transcriptional regulator GlnR
260	SAL0000260	-	rplI	100.0	50S ribosomal protein L9
261	SAL0000261	-	albF	100.0	Putative zinc protease AlbF
262	SAL0000262	7,14	group_6279	100.0	hypothetical protein
263	SAL0000263	-	proX	100.0	Prolyl-tRNA editing protein ProX
264	SAL0000264	-	group_6285	100.0	metal-dependent hydrolase
265	SAL0000265	-	group_6293	100.0	hypothetical protein
266	SAL0000266	-	group_6297	100.0	hypothetical protein
267	SAL0000267	-	lipC	100.0	Spore germination lipase LipC
268	SAL0000268	-	recR	100.0	Recombination protein RecR
269	SAL0000269	-	yidA_4	100.0	Sugar phosphatase YidA
270	SAL0000270	-	paaI	100.0	Acyl-coenzyme A thioesterase PaaI
271	SAL0000271	-	dnaD	100.0	DNA replication protein DnaD
272	SAL0000272	19	rfbC	100.0	putative dTDP-4-dehydrorhamnose 3,5-epimerase
273	SAL0000273	-	cpsB	100.0	Tyrosine-protein phosphatase CpsB
274	SAL0000274	-	group_6325	100.0	SNARE associated Golgi protein
275	SAL0000275	-	ytrA	100.0	HTH-type transcriptional repressor YtrA
276	SAL0000276	6,16	group_6330	100.0	hypothetical protein
277	SAL0000277	-	group_6333	100.0	hypothetical protein
278	SAL0000278	6	group_6335	100.0	hypothetical protein
279	SAL0000279	-	group_6342	100.0	Cupin domain protein
280	SAL0000280	-	group_6343	100.0	putative HTH-type transcriptional regulator
281	SAL0000281	9	group_6344	100.0	putative DNA-binding protein
282	SAL0000282	-	xpt	100.0	Xanthine phosphoribosyltransferase
283	SAL0000283	19	group_6353	100.0	GDSL-like Lipase/Acylhydrolase
284	SAL0000284	-	bglA_2	100.0	6-phospho-beta-glucosidase BglA
285	SAL0000285	-	group_6356	100.0	Aldose 1-epimerase
286	SAL0000286	-	group_6360	100.0	DegV domain-containing protein
287	SAL0000287	-	group_6361	100.0	hypothetical protein
288	SAL0000288	-	cmk	100.0	Cytidylate kinase
289	SAL0000289	-	group_6370	100.0	hypothetical protein
290	SAL0000290	-	rpsU	100.0	30S ribosomal protein S21
291	SAL0000291	-	serB	100.0	Phosphoserine phosphatase
292	SAL0000292	-	gph_2	100.0	Phosphorylated carbohydrates phosphatase
293	SAL0000293	-	group_6377	100.0	hypothetical protein
294	SAL0000294	-	group_6378	100.0	hypothetical protein
295	SAL0000295	6	pspA	100.0	Phosphoserine phosphatase 1
296	SAL0000296	11	group_6380	100.0	hypothetical protein
297	SAL0000297	-	yhbU_2	100.0	putative protease YhbU precursor
298	SAL0000298	-	group_6383	100.0	hypothetical protein
299	SAL0000299	-	group_6384	100.0	hypothetical protein
300	SAL0000300	-	group_6390	100.0	Uracil DNA glycosylase superfamily protein
301	SAL0000301	19	mgsR	100.0	Regulatory protein MgsR
302	SAL0000302	18	group_6392	100.0	CutC-like protein
303	SAL0000303	11	ribU	100.0	Riboflavin transporter RibU
304	SAL0000304	-	scpB	100.0	Segregation and condensation protein B
305	SAL0000305	-	acyP	100.0	Acylphosphatase
306	SAL0000306	8	nrdR	100.0	Transcriptional repressor NrdR
307	SAL0000307	-	group_6405	100.0	hypothetical protein
308	SAL0000308	-	ppaX	100.0	Pyrophosphatase PpaX
309	SAL0000309	12	rluD_1	100.0	Ribosomal large subunit pseudouridine synthase D
310	SAL0000310	-	group_6600	100.0	hypothetical protein
311	SAL0000311	-	ecfA2	100.0	Energy-coupling factor transporter ATP-binding protein EcfA2
312	SAL0000312	-	mecA	100.0	Adapter protein MecA
313	SAL0000313	-	hpdA	100.0	4-hydroxyphenylacetate decarboxylase activating enzyme
314	SAL0000314	-	znuC_2	100.0	High-affinity zinc uptake system ATP-binding protein ZnuC
315	SAL0000315	3,5	rpmD	100.0	50S ribosomal protein L30
316	SAL0000316	3,4,17	rplX	100.0	50S ribosomal protein L24

317	SAL0000317	9	rpsQ	100.0	30S ribosomal protein S17
318	SAL0000318	-	rplP	100.0	50S ribosomal protein L16
319	SAL0000319	-	acpP_2	100.0	Acyl carrier protein
320	SAL0000320	-	group_6749	100.0	Putative protein phosphatase 2C-type
321	SAL0000321	14	ftsL	100.0	Cell division protein FtsL
322	SAL0000322	-	group_6751	100.0	Enterocin A Immunity
323	SAL0000323	3	yidA_3	100.0	Sugar phosphatase YidA
324	SAL0000324	-	rimP	100.0	Ribosome maturation factor RimP
325	SAL0000325	-	group_6755	100.0	Phosphotransferase enzyme family protein
326	SAL0000326	-	group_6756	100.0	HIT-like protein
327	SAL0000327	-	mprA	100.0	Transcriptional repressor MprA
328	SAL0000328	4,7	group_6760	100.0	hypothetical protein
329	SAL0000329	-	acpS	100.0	Holo-[acyl-carrier-protein] synthase
330	SAL0000330	2	rpsF	100.0	30S ribosomal protein S6
331	SAL0000331	11	group_6768	100.0	Colicin V production protein
332	SAL0000332	15	sipU	100.0	Signal peptidase I U
333	SAL0000333	18,21	group_6770	100.0	hypothetical protein
334	SAL0000334	-	group_6771	100.0	hypothetical protein
335	SAL0000335	-	oppF	100.0	Oligopeptide transport ATP-binding protein OppF
336	SAL0000336	2,13	tabA	100.0	Toxin-antitoxin biofilm protein TabA
337	SAL0000337	-	group_6774	100.0	DNA-binding transcriptional activator GutM
338	SAL0000338	-	group_6775	100.0	Endoribonuclease L-PSP
339	SAL0000339	4	groS	100.0	10 kDa chaperonin
340	SAL0000340	-	group_6778	100.0	Cold shock protein CspC
341	SAL0000341	-	arsR_2	100.0	Arsenical resistance operon repressor
342	SAL0000342	-	group_6802	100.0	hypothetical protein
343	SAL0000343	6,8	rlmH	100.0	Ribosomal RNA large subunit methyltransferase H
344	SAL0000344	-	group_6804	100.0	Septum formation initiator
345	SAL0000345	-	group_6805	100.0	GDSL-like Lipase/Acylhydrolase
346	SAL0000346	-	group_6814	100.0	hypothetical protein
347	SAL0000347	-	group_6815	100.0	hypothetical protein
348	SAL0000348	-	group_6816	100.0	hypothetical protein
349	SAL0000349	-	group_6817	100.0	hypothetical protein
350	SAL0000350	-	group_6819	100.0	hypothetical protein
351	SAL0000351	-	group_6822	100.0	hypothetical protein
352	SAL0000352	2,15	nudG	100.0	CTP pyrophosphohydrolase
353	SAL0000353	-	group_6826	100.0	D-Ala-teichoic acid biosynthesis protein
354	SAL0000354	19	group_6828	100.0	hypothetical protein
355	SAL0000355	-	group_6829	100.0	Acyl-ACP thioesterase
356	SAL0000356	-	group_6832	100.0	hypothetical protein
357	SAL0000357	-	plsY	100.0	Glycerol-3-phosphate acyltransferase
358	SAL0000358	-	group_6834	100.0	hypothetical protein
359	SAL0000359	-	panT	100.0	Pantothenic acid transporter PanT
360	SAL0000360	-	nrdD_1	100.0	Anaerobic ribonucleoside-triphosphate reductase
361	SAL0000361	18	maa	100.0	Maltose O-acetyltransferase
362	SAL0000362	-	group_6839	100.0	hypothetical protein
363	SAL0000363	-	group_6840	100.0	hypothetical protein
364	SAL0000364	-	azr_4	100.0	NADPH azoreductase
365	SAL0000365	-	group_6844	100.0	hypothetical protein
366	SAL0000366	-	yhhX	100.0	putative oxidoreductase YhhX
367	SAL0000367	-	folB	100.0	Dihydroneopterin aldolase
368	SAL0000368	1	immR_1	100.0	HTH-type transcriptional regulator ImmR
369	SAL0000369	-	group_6853	100.0	hypothetical protein
370	SAL0000370	-	group_6855	100.0	Muramidase-2 precursor
371	SAL0000371	-	rimM	100.0	Ribosome maturation factor RimM
372	SAL0000372	1	rpsP	100.0	30S ribosomal protein S16
373	SAL0000373	-	pyrB	100.0	Aspartate carbamoyltransferase
374	SAL0000374	-	rpmI	100.0	50S ribosomal protein L35
375	SAL0000375	-	atpF	100.0	ATP synthase subunit b
376	SAL0000376	-	rplS	100.0	50S ribosomal protein L19
377	SAL0000377	-	group_6865	100.0	hypothetical protein
378	SAL0000378	-	group_6866	100.0	putative metallo-hydrolase
379	SAL0000379	-	glpQ1	100.0	putative glycerophosphoryl diester phosphodiesterase 1
380	SAL0000380	-	group_6868	100.0	Glycosyl hydrolases family 25
381	SAL0000381	20	thiT	100.0	Thiamine transporter ThiT
382	SAL0000382	12	group_6870	100.0	hypothetical protein

383	SAL0000383	-	phoP	100.0	Alkaline phosphatase synthesis transcriptional regulatory protein PhoP
384	SAL0000384	3	group_6873	100.0	Glyoxalase-like domain protein
385	SAL0000385	12	yycJ	100.0	Putative metallo-hydrolase YycJ
386	SAL0000386	-	group_6876	100.0	cellobiose phosphotransferase system IIB component
387	SAL0000387	-	gloA	100.0	Lactoylglutathione lyase
388	SAL0000388	14	group_6883	100.0	inosine 5'-monophosphate dehydrogenase
389	SAL0000389	9	group_6884	100.0	hypothetical protein
390	SAL0000390	-	clpP	100.0	ATP-dependent Clp protease proteolytic subunit
391	SAL0000391	-	group_6887	100.0	hypothetical protein
392	SAL0000392	-	group_6889	100.0	Putative membrane protein insertion efficiency factor
393	SAL0000393	-	group_6890	100.0	hypothetical protein
394	SAL0000394	-	rsfS	100.0	Ribosomal silencing factor RsfS
395	SAL0000395	-	group_6892	100.0	GTPase YlqF
396	SAL0000396	-	group_6917	100.0	hypothetical protein
397	SAL0000397	-	yugI	100.0	General stress protein 13
398	SAL0000398	-	group_7149	100.0	YtxH-like protein
399	SAL0000399	-	yjiR	100.0	putative HTH-type transcriptional regulator YjiR
400	SAL0000400	-	group_7206	100.0	hypothetical protein
401	SAL0000401	-	rplC	100.0	50S ribosomal protein L3
402	SAL0000402	-	group_979	100.0	hypothetical protein

Supplementary File S3.D. Genetic relatedness tree from wgMLST (36 strains studied; no reference strains included).

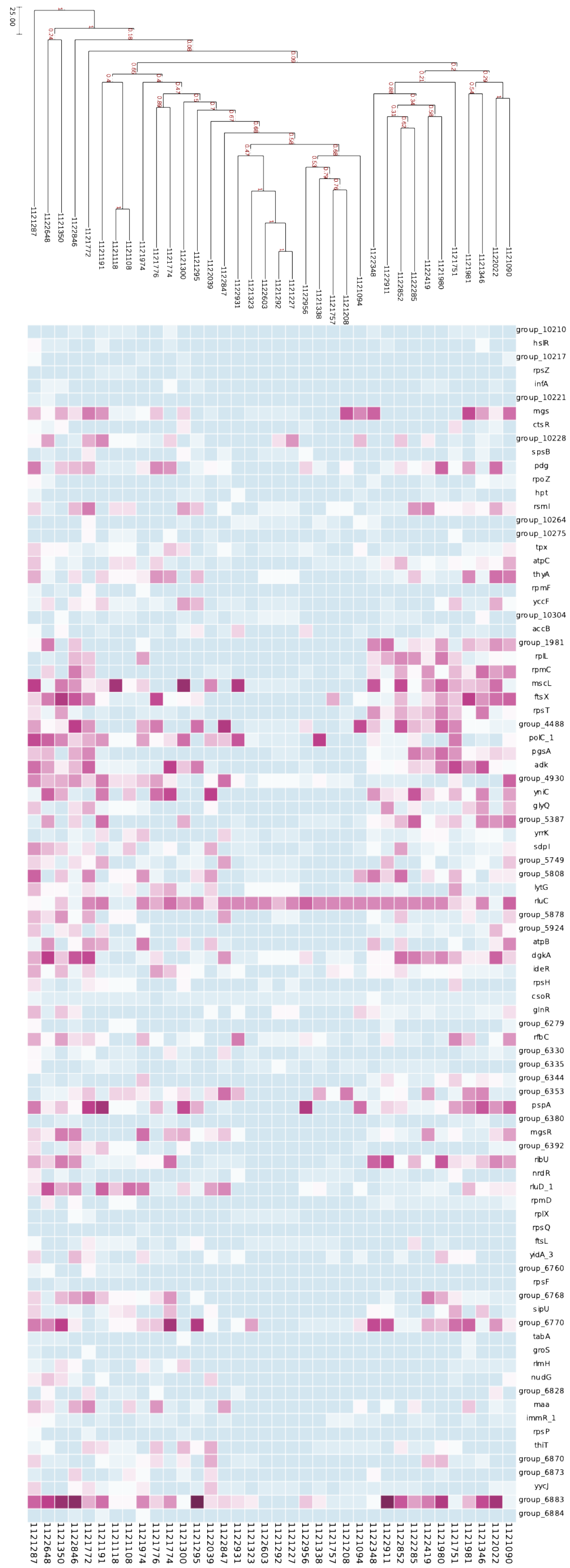

Supplement: Supplementary file 1 [file antibiotics-14-01059-s001.zip › antibiotics-3895307-supplementary/Supplementary File S3.pdf]
