# Supplementary material for: Genomic Characterization and Antimicrobial Resistance Profile of Streptococcus uberis Strains Isolated from Cows with Mastitis from Northwestern Spain
Source: Antibiotics (Basel). 2025 Oct 23;14(11):1059. doi: 10.3390/antibiotics14111059 (PMC12649216; doi:10.3390/antibiotics14111059)
Supplement: Supplementary file 1 [file antibiotics-14-01059-s001.zip › antibiotics-3895307-supplementary/Supplementary File S4.pdf]

Supplementary File S4. Predicted resistance phenotype to antibiotics from the *S. uberis* strains studied.

Strains Code	Predicted resistance phenotype
1121090	Streptomycin;Lincomycin;Clindamycin;Dalfopristin;Pristinamycin_IIA;Virginiamycin_M;Tiamulin
1121094	Erythromycin;Lincomycin;Clindamycin;Quinupristin;Pristinamycin_IA;Virginiamycin_S
1121108	Lincomycin
1121118	Lincomycin
1121191	Lincomycin
1121208	Erythromycin;Spiramycin;Telithromycin
1121227	Doxycycline;Tetracycline;Minocycline;Streptomycin;Lincomycin;Clindamycin;Dalfopristin;Pristinamycin_IIA; Virginiamycin_M;Tiamulin
1121287	Erythromycin;Lincomycin;Clindamycin;Quinupristin;Pristinamycin_IA;Virginiamycin_S
1121292	Streptomycin;Lincomycin;Clindamycin;Dalfopristin;Pristinamycin_IIA;Virginiamycin_M;Tiamulin;Doxycycline; Tetracycline;Minocycline
1121295	Erythromycin;Lincomycin;Clindamycin;Quinupristin;Pristinamycin_IA;Virginiamycin_S
1121300	-
1121323	Streptomycin;Lincomycin;Clindamycin;Dalfopristin;Pristinamycin_IIA;Virginiamycin_M;Tiamulin;Doxycycline; Tetracycline;Minocycline
1121338	-
1121346	Doxycycline;Tetracycline;Minocycline;Erythromycin;Lincomycin;Clindamycin;Quinupristin;Pristinamycin_IA; Virginiamycin_S;Amikacin;Streptomycin
1121350	Doxycycline;Tetracycline;Minocycline;Erythromycin;Lincomycin;Clindamycin;Quinupristin;Pristinamycin_IA; Virginiamycin_S;Amikacin;Streptomycin
1121751	Streptomycin;Lincomycin;Clindamycin;Dalfopristin;Pristinamycin_IIA;Virginiamycin_M;Tiamulin
1121757	Doxycycline;Tetracycline;Minocycline;Erythromycin;Spiramycin;Telithromycin;Streptomycin;Lincomycin; Clindamycin;Dalfopristin;Pristinamycin_IIA;Virginiamycin_M;Tiamulin
1121772	Doxycycline;Tetracycline;Minocycline;Streptomycin;Lincomycin;Clindamycin;Dalfopristin;Pristinamycin_IIA; Virginiamycin_M;Tiamulin
1121774	Streptomycin;Lincomycin;Clindamycin;Dalfopristin;Pristinamycin_IIA;Virginiamycin_M;Tiamulin
1121776	Streptomycin; Lincomycin;Clindamycin;Dalfopristin;Pristinamycin_IIA;Virginiamycin_M;Tiamulin; Erythromycin;Quinupristin;Pristinamycin_IA;Virginiamycin_S
1121974	Doxycycline;Tetracycline;Minocycline;Erythromycin;Lincomycin;Clindamycin;Quinupristin;Pristinamycin_IA; Virginiamycin_S;Streptomycin
1121980	Doxycycline;Tetracycline;Minocycline;Streptomycin;Amikacin;Erythromycin;Lincomycin;Clindamycin; Quinupristin;Pristinamycin_IA;Virginiamycin_S
1121981	-
1122022	Erythromycin;Lincomycin;Clindamycin;Quinupristin;Pristinamycin_IA;Virginiamycin_S;Amikacin;Streptomycin; Dalfopristin;Pristinamycin_IIA;Virginiamycin_M;Tiamulin
1122039	Streptomycin;Lincomycin;Clindamycin;Dalfopristin;Pristinamycin_IIA;Virginiamycin_M;Tiamulin;Doxycycline; Tetracycline;Minocycline
1122285	Amikacin;Streptomycin;Doxycycline;Tetracycline;Minocycline;Erythromycin;Lincomycin;Clindamycin; Quinupristin;Pristinamycin_IA;Virginiamycin_S;alfopristin;Pristinamycin_IIA;Virginiamycin_M;Tiamulin
1122348	Streptomycin;Doxycycline;Tetracycline;Minocycline;Erythromycin;Lincomycin;Clindamycin;Quinupristin; Pristinamycin_IA;Virginiamycin_S
1122419	Streptomycin;Lincomycin;Clindamycin;Dalfopristin;Pristinamycin_IIA;Virginiamycin_M;Tiamulin
1122603	Doxycycline;Tetracycline;Minocycline;Streptomycin;Lincomycin;Clindamycin;Dalfopristin;Pristinamycin_IIA; Virginiamycin_M;Tiamulin
1122648	Erythromycin;Lincomycin;Clindamycin;Quinupristin;Pristinamycin_IA;Virginiamycin_S
1122846	-
1122847	Streptomycin;Lincomycin;Clindamycin;Dalfopristin;Pristinamycin_IIA;Virginiamycin_M;Tiamulin
1122852	Doxycycline;Tetracycline;Minocycline;Streptomycin;Lincomycin;Clindamycin;Dalfopristin;Pristinamycin_IIA; Virginiamycin_M;Tiamulin
1122911	Doxycycline;Tetracycline;Minocycline;Streptomycin;Lincomycin;Clindamycin;Dalfopristin;Pristinamycin_IIA; Virginiamycin_M;Tiamulin;Erythromycin;Spiramycin;Telithromycin;Streptomycin
1122931	Doxycycline;Tetracycline;Minocycline;Streptomycin;Lincomycin;Clindamycin;Dalfopristin;Pristinamycin_IIA; Virginiamycin_M;Tiamulin
1122956	Erythromycin;Lincomycin;Clindamycin;Quinupristin;Pristinamycin_IA;Virginiamycin_S
