# Supplementary material for: Genomic Characterization and Antimicrobial Resistance Profile of Streptococcus uberis Strains Isolated from Cows with Mastitis from Northwestern Spain
Source: Antibiotics (Basel). 2025 Oct 23;14(11):1059. doi: 10.3390/antibiotics14111059 (PMC12649216; doi:10.3390/antibiotics14111059)
Supplement: Supplementary file 1 [file antibiotics-14-01059-s001.zip › antibiotics-3895307-supplementary/Supplementary File S5.pdf]

Supplementary File S5.A. *S. uberis* strains showing results from the PlasmidFinder tool.

Strain	Plasmid	Identity (%)	Contig	Position in contig
1121227	repUS43	99.75	Utg1016	69816..71021
1121292	repUS43	99.75	Utg1062	1133845..1135050
1121323	repUS43	99.75	Utg668	412234..413439
1121757	repUS43	99.83	Utg554	1041359..1042564
1121772	repUS43	99.59	Utg1090	811760..812964
1122348	repUS43	99.92	Utg626	1387319..1388524
1122603	repUS43	99.75	Utg848	963002..964207
1122852	repUS43	99.83	Utg3174	625896..627101
1122911	repUS43	99.92	Utg600	91321..92526
1122931	repUS43	100	Utg548	1676993..1678198

Targeted loci: Rep1, NT_Rep, RepA_N, Rep2, Rep_trans, Inc18, Rep3, RepL. All strains targeted the locus Rep_trans. GenBank accession number of plasmid repUS43: CP003584. Query/Template Length: 1206/1206.

Supplementary File S5.B. Additional information related to the virulence factors (VF) that contribute to the pathogenicity of *S. uberis*. Different open reading frames (orf) are shown for each gene.

**VF class**	**Virulence factors**	**Related genes**	**Prediction (strains)**						
			**1121090**	**1121094**	**1121108**	**1121118**	**1121191**	**1121208**	**1121227**
Adherence	Agglutinin receptor	Undetermined							
	Fibronectin-binding proteins	fbp54	Orf01520	Orf01082	Orf00055	Orf01116	Orf01219	Orf00022	Orf00189
	Laminin-binding proteins	lmb	Orf01827	Orf00825	Orf00354		Orf01563	Orf00265	Orf01474, orf01854
	Streptococcal lipoprotein rotamase A	slrA		Orf00414			Orf01940	-	Orf01135
	Streptococcal plasmid receptor/GAPDH	plr/gapA		Orf01594		Orf00548	Orf00315	Orf01351	Orf00721
	rlrA islet	srtC							
Enzyme	Hyaluronidase	hyIB			Orf02117	Orf00095, orf00866			Orf00420
	Streptococcal enolase	eno	Orf02102	Orf00594	Orf00623	Orf01582	Orf01785	Orf00487	Orf00941
Immune evasion	Capsule	Undetermined	Orf00854, orf00858, orf00859, orf01609-12, orf01623-25, orf01627, orf02044, orf02050	Orf00641, orf00646, orf00987-90, orf00998-1003, orf01527, orf01528, orf01532	Orf00156, orf00157-59, orf00172, orf00563, orf01651, orf01652, orf01657	Orf00481, orf00483, orf00487, orf01193-97, orf01208, orf01535	Orf00378, orf01293-98, orf01306, orf01308, orf01737, orf01742	Orf00097-101, orf00113, orf00434, orf00438, orf00440, orf00444, orf00445, orf01284, orf01286, orf01290	Orf00093, orf00095, orf00103-8, orf00784, orf00788, orf00789, orf02030, orf02035
	Polysaccharide capsule (Bacillus)	- galE			Orf00171		Orf01307	Orf00103	Orf00094
	Exopolysaccharide (Haemophilus)	galE							
Immunoreactive antigen	Rib	rib							Orf01471
Iron uptake									
Manganese uptake	Pneumococcal surface antigen A / Metal binding protein SloC	psaA	Orfo2333	Orf00411	Orf00834	Orf01748	Orf01943	Orf00661	Orf01139
Protease	C3-degrading protease	cppA	Orf00725	Orf01630	Orf01782	Orf00576	Orf00252	Orf01403	Orf00676
	C5a peptidase	scpA/scpB	Orf01462	Orf01129		Orf01071	Orf01173	Orf01871	Orf00233
	Serine protease	htrA/degP	Orf01126	Orf01372	Orf01464	Orf00327	Orf00551	Orf01135	Orf01700
	Trigger factor	tig/ropA	-	Orf00008	Orf01071	Orf01943	Orf00579	Orf00833	Orf01346

Superantigen									
Toxin									
Cell surface components	Trehalose-recycling ABC transporter (Mycobacterium)	sugC				Orf00170	Orf00802		
Serum resistance and immune evasion	LPS (Francisella)	wbtP							

VF class	Virulence factors	Related genes	Prediction (strains)												
			**1121292**	**1121295**	**1121300**	**1121323**	**1121338**	**1121350**	**1121751**	**1121757**	**1121772**	**1121776**	**1121974**	**1121980**	**1121981**
Adherence	Agglutinin receptor	Undetermined						Orf02040, orf02041					Orf00309, orf00311	Orf01029	
	Fibronectin-binding proteins	fbp54	Orf01690	Orf00733	Orf01773	Orf01111	Orf00401	Orf01323	Orf01955	Orf00875	Orf01654	Orf01162	Orf00452	Orf01178	Orf01651
	Laminin-binding proteins	lmb	Orf00708, orf01440	-	Orf01523	Orf00873	Orf00643	-	Orf02268	Orf00632	Orf02093, orf03133	Orf01446	Orf00747	Orf01474	Orf01967
	Streptococcal lipoprotein rotamase A		Orf01032	-	-	-	-	-	-	-	-	-	Orf01214	Orf02006	Orf02483
	Streptococcal plasmid receptor/GAPDH	plr/gapA	Orf00198	Orf00204	Orf00424	Orf01729	Orf01529	Orf02100	-	Orf01461	Orf00810	Orf00570	Orf02094	Orf00475	-
	rlrA islet	srtC			Orf00848										
Enzyme	Hyaluronidase	hyIB	Orf01981, orf01982	-	-	Orf01394	-	-	-	-	Orf01277, orf01278	-	-	Orf00822	-
	Streptococcal enolase	eno	Orf01221	Orf01186	Orf01286	Orf00655	Orf00858	Orf00031	-	Orf00415	-	Orf01678	Orf01015	Orf01740	Orf02261
Immune evasion	Capsule	Undetermined	Orf00260, orf00264, orf00265, orf01264, orf01269, orf01595, orf01597, orf01605-11	Orf00110, orf00111, orf00115, orf00810-16, orf00826, orf01139, orf01143	Orf00360, orf00362, orf00366, orf00457, orf01330, orf01331, orf01335, orf01337, orf01342, orf01679, orf01689-95	Orf00697, orf00702, orf01026, orf01036-41, orf01789, orf01793, orf01794	Orf00477-82, orf00491, orf00812, orf00817, orf01463, orf01465, orf01469	Orf00080, orf00081, orf00085, orf00088, orf00092, orf01226, orf01236-38, orf01240-43, orf02161	Orf01208, orf01212, orf01213, orf02061-64, orf02067, orf02080, orf02484, orf02491	Orf00456, orf00461, orf00797-800, orf01522, orf01526, orf01527	Orf00667, orf00668, orf00674, orf01863, orf02359	Orf00324, orf00328, orf00329, orf01235-41, orf01617, orf01622	Orf00550, orf00551, orf00554, orf00558, orf00571, orf00947, orf02017, orf02018, orf02024	Orf01274-6, orf01291, orf01683, orf01691, orf02441, orf02442, orf02446	Orf00876, orf00880, orf00881, orf01754-7, orf01760, orf02198, orf02205
	Polysaccharide capsule (Bacillus)	- galE	Orf01596					Orf01227		-					Orf01280 Orf01290
	Exopolysaccharide (Haemophilus)	galE								Orf00784					
Immunoreactive antigen	Rib	rib												Orf00166	
Iron uptake															
Manganese uptake	Pneumococcal surface antigen A / Metal binding protein SloC	psaA	Orf01029	Orf01353	Orf01106	Orf00469	Orf01085	Orf00415	Orf00248	Orf00232	Orf02682	Orf01851	Orf01217	Orf02009	Orf02486
Protease	C3-degrading protease	cppA	Orf00154	Orf00235	Orf00497	Orf01687	Orf01566	Orf01049	Orf01056	Orf01408	Orf00895	Orf00642	Orf02135	Orf00517	Orf00724
	C5a peptidase	scpA/scpB	Orf01736	Orf00687	Orf01818	Orf01155	Orf00356	-	-	Orf00920	Orf01586	Orf01117	Orf00398	-	Orf01591
	Serine protease	htrA/degP	Orf00484	Orf01810	Orf00190	Orf01971	Orf01260	Orf01510	Orf01422	Orf01673	Orf00361	Orf00474	Orf01820	Orf02246	Orf01099
	Trigger factor	tig/ropA	Orf00830	Orf01524	Orf01985	Orf00290	Orf00110	Orf01733	-	Orf00050	Orf02953	Orf00499	Orf01433	-	-

Superantigen															
Toxin															
Cell surface components	Trehalose-recycling ABC transporter (Mycobacterium)	sugC		Orf01654	Orf00790			Orf01924		Orf01839		Orf00079	Orf01618	Orf00361	
Serum resistance and immune evasion	LPS (Francisella)	wbtP								Orf00795					

VF class	Virulence factors	Related genes	Prediction (strains)											
			**1122022**	**1122039**	**1122285**	**1122348**	**1122419**	**1122603**	**1122648**	**1122846**	**1122847**	**1122852**	**1122911**	**1122931**	**1122956**
Adherence	Agglutinin receptor	Undetermined			Orf01945	Orf00333, orf00335, orf00336									
	Fibronectin-binding proteins	fbp54	Orf01302	Orf00916	Orf01798	Orf00204-5	Orf02275	Orf00476	Orf00892	Orf00169	Orf01604	Orf01088	orf00327	orf00084	orf01490
	Laminin-binding proteins	lmb	Orf00999	Orf01209	Orf01504	Orf01325, orf01695	Orf00302	Orf00723	Orf01212	Orf02290	Orf01362	Orf02350	Orf00632; orf01518	orf01782	orf01741
	Streptococcal lipoprotein rotamase A		Orf00507	-	-	-	Orf00783	Orf01131	-	Orf01791	-	Orf01334	Orf01149		
	Streptococcal plasmid receptor/GAPDH	plr/gapA	-	Orf00306	-	-	-	Orf01857	-	-	Orf00177	-	-	orf00589	orf00321
	rlrA islet	srtC													
Enzyme	Hyaluronidase	hyIB	-	Orf00640	-	-	Orf01979-80	Orf00249	-	-	-	Orf00754, orf00755	Orf00003; orf00004		
	Streptococcal enolase	eno	Orf00730	Orf01489	Orf01231	Orf01935	Orf00576	Orf00944	Orf01502	Orf02004	Orf01148	Orf01864	Orf00932	orf01535	orf01973
Immune evasion	Capsule	Undetermined	Orf00776, orf00782, orf01196, orf01197, orf01201-3, orf02132, orf02138	Orf00224. Orf00225, orf00229, orf01010-4, orf01027, orf01432, orf01437	Orf00077, orf00081-2, orf01283, orf01288, orf01699, orf01702-4	Orf00122-4, orf00940, orf00945-6, orf01879, orf01884	Orf00104-7, orf00110, orf00122, orf00518, orf00522, orf00525, orf01678, orf01682-3	Orf00544-48, orf00895, orf00899, orf01791, orf01793, orf01797	Orf00083, orf00993-96, orf00998, orf01013, orf01442, orf01448	Orf00044 – 48, orf00053, orf00056, orf00059-62, orf00937, orf00941-42, orf02054, orf02061	Orf00237, orf00241, orf00242, orf01189, orf01194, orf01511, orf01519-25	0rf00263, orf00271, orf01799, orf01807, orf02139 – orf02142, orf02146, orf02160	Orf00433 – orf00436; orf00439; orf0040; orf00449; orf00871; orf00878; orf02082; orf02083; orf02087	orf00005 - orf00011; orf00652; orf00656; orf00657; orf01577; orf01578; orf01583; orf01590; orf01530	orf00382; orf00386; orf00388; orf01569 - orf01575; orf01584 - orf01586; orf01924; orf01929
	Polysaccharide capsule (Bacillus)	-	-	-	-	Orf00117	-		Orf01000	-	Orf01512			orf01931	
	Exopolysaccharide (Haemophilus)	galE	-	-	-	-	-	Orf00562							
Immunoreactive antigen	Rib	rib			Orf00630	Orf01331									
Iron uptake															
Manganese uptake	Pneumococcal surface antigen A / Metal binding protein SloC	psaA	Orf00504	Orf01697	Orf01024	Orf02112	Orf00786	Orf01134	Orf01718	Orf01788	Orf00974	Orf01331	Orf01152	orf01357	orf01042
Protease	C3-degrading protease	cppA	Orf02011	Orf00353	Orf02494	Orf00824	Orf01568	Orf01900	Orf00235	Orf00785	Orf00141	Orf00418	Orf02263	orf00545	orf00268
	C5a peptidase	scpA/scpB	Orf01362	Orf00859	-	-	Orf02217	Orf00431	-	-	Orf01647	-	Orf00269	orf00129	orf01443
	Serine protease	htrA/degP	Orf02345	Orf00023	Orf00263	Orf01102	orf01883	Orf01628	Orf02417	Orf01167	Orf00421	Orf00059	Orf01888	orf00827	orf00544
	Trigger factor	tig/ropA	-	Orf01901	-	-	-	Orf01328	-	-	Orf00792	-		orf01188	orf00867

Superantigen															
Toxin															
Cell surface components	Trehalose-recycling ABC transporter (Mycobacterium)	sugC								Orf01380					
Serum resistance and immune evasion	LPS (Francisella)	wbtP						Orf00550							

Supplementary File S5.C. PathogenFinder tool. All the strains studied were predicted to be human pathogens.

Strain	Input sequence information				Results		
	Seq.	Total bpp	Longest seq.	Shortest seq.	P	Genome coverage	F/M
1121090	2452	574183	2931	31	0.82	0.41	9/10
1121094	1971	554083	1484	31	0.89	0.25	5/5
1121108	2326	582982	2977	30	0.83	0.39	8/9
1121118	2260	636059	2977	31	0.91	0.75	17/17
1121191	2061	608347	3101	31	0.91	0.29	6/6
1121208	1878	559889	1484	31	0.92	0.32	6/6
1121227	2023	575881	1796	30	0.89	0.49	10/10
1121287	2315	667003	5315	30	0.89	0.39	9/9
1121292	2136	608511	1796	30	0.88	0.42	9/9
1121295	1970	584464	1484	30	0.89	0.30	6/6
1121300	1961	572893	1490	31	0.84	0.51	9/10
1121323	2011	587865	1796	32	0.80	0.40	7/8
1121338	1976	574172	1556	30	0.89	0.51	10/10
1121346	2397	582124	1370	30	0.86	0.83	19/20
1121350	2174	582603	1983	30	0.91	0.87	19/19
1121751	2516	596770	2094	32	0.81	0.44	10/11
1121757	1901	566012	1484	31	0.78	0.37	6/7
1121772	3088	610779	1580	30	0.88	0.78	24/24
1121774	1933	586186	2792	31	0.87	0.47	9/9
1121776	2061	598362	1583	30	0.78	0.39	7/8
1121974	2449	603142	1226	30	0.89	1.10	27/27
1121980	2460	599781	1632	30	0.89	0.85	21/21
1121981	2603	585689	1370	30	0.80	0.35	8/9
1122022	2489	589757	2267	30	0.80	0.40	9/10
1122039	2308	558702	2392	31	0.91	0.43	10/10
1122285	2568	622820	1632	30	0.89	0.86	22/22
1122348	2600	624434	1907	30	0.90	0.96	25/25
1122419	2290	551627	1370	30	0.82	0.48	10/11
1122603	1887	554706	1796	31	0.82	0.48	8/9
1122648	2503	582853	2959	30	0.83	0.36	8/9
1122846	2395	567693	2961	30	0.75	0.42	8/10
1122847	1905	572217	1758	31	0.87	0.42	8/8
1122852	2550	568958	2392	31	0.82	0.43	10/11
1122911	2516	598115	1550	30	0.80	0.48	11/12
1122931	1921	562884	1466	30	0.91	0.52	10/10

1122956	2043	568166	1534	32	0.84	0.49	9/10

P: Probability of being a human pathogen. A probability value associated with the prediction. Genome coverage (%): The percentage of input sequences that were used to compute the final prediction. F/M: Matched pathogenic families (the number of input sequences that matched pathogenic protein families in the family's database)/Total matches (the number of input sequences that matched pathogenic and nonpathogenic protein families in the family's database). Seq.: sequences.
